# Structural and phylogenetic analysis of a conserved actinobacteria-specific protein (ASP1; SCO1997) from *Streptomyces coelicolor*

**DOI:** 10.1186/1472-6807-9-40

**Published:** 2009-06-10

**Authors:** Beile Gao, Seiji Sugiman-Marangos, Murray S Junop, Radhey S Gupta

**Affiliations:** 1 Department of Biochemistry and Biomedical Science, McMaster University, Hamilton, L8N3Z5, Canada; 2 Michael G. DeGroote Institute for Infectious Disease Research, McMaster University, Hamilton, L8N3Z5, Canada

## Abstract

**Background:**

The Actinobacteria phylum represents one of the largest and most diverse groups of bacteria, encompassing many important and well-characterized organisms including *Streptomyces, Bifidobacterium, Corynebacterium *and *Mycobacterium*. Members of this phylum are remarkably diverse in terms of life cycle, morphology, physiology and ecology. Recent comparative genomic analysis of 19 actinobacterial species determined that only 5 genes of unknown function uniquely define this large phylum [1]. The cellular functions of these actinobacteria-specific proteins (ASP) are not known.

**Results:**

Here we report the first characterization of one of the 5 actinobacteria-specific proteins, ASP1 (Gene ID: SCO1997) from *Streptomyces coelicolor*. The X-ray crystal structure of ASP1 was determined at 2.2 Ǻ. The overall structure of ASP1 retains a similar fold to the large NP-1 family of nucleoside phosphorylase enzymes; however, the function is not related. Further comparative analysis revealed two regions expected to be important for protein function: a central, divalent metal ion binding pore, and a highly conserved elbow shaped helical region at the C-terminus. Sequence analyses revealed that ASP1 is paralogous to another actinobacteria-specific protein ASP2 (SCO1662 from *S. coelicolor*) and that both proteins likely carry out similar function.

**Conclusion:**

Our structural data in combination with sequence analysis supports the idea that two of the 5 actinobacteria-specific proteins, ASP1 and ASP2, mediate similar function. This function is predicted to be novel since the structures of these proteins do not match any known protein with or without known function. Our results suggest that this function could involve divalent metal ion binding/transport.

## Background

Actinobacteria constitute one of the main phyla within the *Bacteria *and they are highly diverse in terms of their morphology, physiology and ecology [2-5]. These bacteria are characterized by high G+C content (greater than 55 mol%) [3,4] and a monoderm cell structure (i.e. bounded by a single membrane)[6,7]. They include *Streptomyces*, the major antibiotic producers in the pharmaceutical industry as well as many important human, animal and plant pathogens, such as *Mycobacterium, Tropheryma, Nocardia, Propionibacterium, Leifsonia*, etc. However, except for their clustering in the 16S rRNA tree, no molecular, biochemical or physiological characteristics are known that can clearly distinguish species belonging to the phylum *Actinobacteria *from other bacteria [8,9].

Comparative analyses of genomic sequences are enabling identification of novel genetic characteristics that are unique to different groups of bacteria. Large numbers of proteins and conserved indels (inserts and deletions) that are specific for various prokaryotic groups such as Archaea, Chlamydiae, Bacteriodetes-Chlorobi, Proteobacteria, etc. have been identified [10-14]. Our recent comparative genomic studies on available actinobacterial genomes have identified a large number of proteins that are either specific for all actinobacterial species or certain subgroups within this phylum [1]. Blast searches with these proteins show no significant hits or similarity to any other protein in the databases. These proteins thus provide novel and useful molecular markers for this diverse group of bacteria [1]. Among these actinobacteria-specific proteins, five proteins (corresponding to ML1009, ML1306, ML1029, ML0257 and ML0642 from the genome of *Mycobacterium leprae *TN) were found in every sequenced actinobacterial species [1] including those from the deepest branch *Rubrobacter xylanophilus *and also from intracellular pathogens such as *Tropheryma whipplei *which have highly reduced genomes [9,15]. All five of these proteins are conserved within actinobacteria but have no known function. These five actinobacteria-specific proteins are referred to in this work as ASP-1, 2, 3, 4 and 5. The simplest and most logical explanation for the persistence of these proteins in only actinobacteria is that their genes evolved only once in a common ancestor of all actinobacteria and were subsequently passed on to all their decedents. So these genes/proteins provide among the very few molecular characteristics known that are distinctive of the *Actinobacteria *phylum [1,8,16,17]. In view of their actinobacteria-specificity, it is of great interest to determine the cellular functions of these proteins and the cellular processes in which they participate. These studies are expected to provide novel insights into biochemical processes and physiological characteristics that are unique to actinobacteria.

In an attempt to gain insight into the cellular functions of these proteins, we have initiated structural work on these 5 actinobacteria-specific proteins. We report here the crystal structure of SCO1997 from *S. coelicolor*, which corresponds to the protein ML1009 from *M. leprae *(ASP1) [1]. Structural and phylogenetic analysis indicates that although ASP1 retains a similar overall fold compared to members of the hydrolase superfamily such as purine nucleoside phosphorylase, the active site region and therefore function of ASP1 are distinct [18,19]. Comparison of the most highly conserved sequences of ASP1 from different actinobacteria with their positions in the crystal structure reveals a potential role for ASP1 in binding and transport of divalent metal ion. Interestingly, additional sequence and structural analyses show that another actinobacteria-specific protein ASP2 (SCO1662; ML1306) is evolutionarily and functionally related to ASP1 [1].

## Results and discussion

### Crystal Structure of ASP1 from *S. coelicolor*

The protein ASP-1 is of hypothetical or unknown function. The genes involved in related functions (e.g. those that are part of an operon) are generally clustered in various species or closely related species. Thus, genetic linkage studies can often provide valuable clues regarding possible cellular function of a given gene/protein [20-22]. Hence, we have examined the neighboring genes of ASP1 in various sequenced actinobacteria. The genes flanking ASP1 in different actinobacterial genomes are either of unknown function or perform unrelated functions. The information for these flanking genes is presented in the Additional file 1 and it provides no clue regarding the possible cellular function of this protein.

To gain insight into the cellular function of ASP1, we have cloned, expressed and crystallized the gene for this protein from *S. coelicolor *A3(2) [1]. The Gene ID and accession numbers of this protein from other actinobacterial genomes are provided in the Additional file 2. The crystal structure of full length ASP1 was determined using Seleno-methionine (SeMet) derivatized ASP1 and single anomalous diffraction (SAD) techniques. The final model was refined with native data (2.2 Ǻ) to R and R_free _values of 17.4% and 23.4%, respectively. The structure of ASP1 contained three regions that were unable to be traced into electron density and therefore not included in the final model. These disordered regions included the first 2 residues at the N-terminus, the last 36 C-terminal residues (amino acids 277–312) as well as a short loop region encompassing residues 168–172. A complete list of data collection and model refinement statistics can be found in Table 1.

**Table 1:** Crystallographic data and model refinement statistics.

	Native^a^	Se-SAD^a^
**Data collection**		
Space group	*I*23	*I*23
Cell dimensions		
*a*, *b*, *c *(Å)	135.1, 135.1, 135.1	135.4, 135.4, 135.4
α, β, γ (°)	90, 90, 90	90, 90, 90
Wavelength	1.1000	0.9794
Resolution (Å)^b^	50.0–2.2 (2.24–2.2)	50.0–2.5 (2.59–2.5)
*R*_merge _(%)^b^	6.7 (42.4)	9.7 (43.7)
*I*/σ (*I*)^b^	39.1 (8.6)	18.7 (6.3)
Completeness (%)^b^	99.3 (100.0)	98.6 (100.0)
Redundancy^b^	21.6 (22.0)	22.7 (23.0)
**Refinement**		
Resolution (Å)	50.0–2.2	
No. reflections	19,088	
*R*_work_/*R*_free_	17.4%/23.4%	
No. atoms		
Protein	2085	
Ligand/ion	2	
Water	272	
*B*-factors	34.3	
R.m.s deviations		
Bond lengths (Å)	0.03	
Bond angles (°)	2.4	

^a ^One crystal was used for data collection.

^b ^Values in parentheses are for highest-resolution shell.

**Figure 1:**
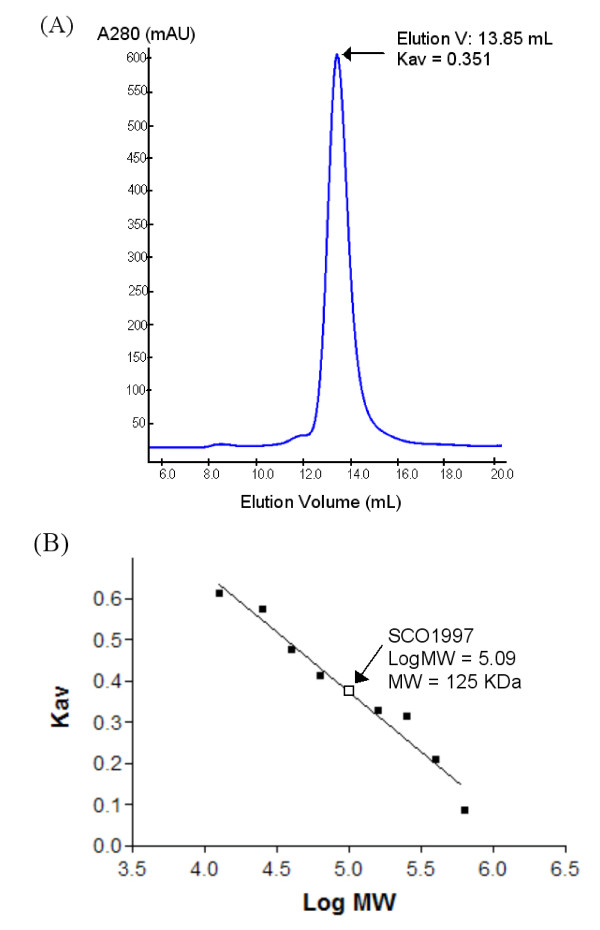
**Size exclusion chromatography of ASP1**. (A) Size exclusion chromatographic analysis of full length ASP1 at 5 mg/mL. A single peak was eluted at 13.8 mL, consistent with the expected elution volume of a roughly globular ~125 kDa protein. (B) Standard Curve for Calibration of S200 size exclusion column.

Crystals grew in space group *I*23 and contained a single copy of ASP1 in the asymmetric unit. Upon inspection of crystallographic packing interactions it appeared that ASP1 might exist as a trimer. The amount of surface area buried through the formation of an ASP1 trimer is significant at 7560 Ǻ^2^. As well, when analyzed by size exclusion chromatography (Figure 1), ASP1 eluted with a Stokes radius consistent with a molecular mass equivalent to ~125 kDa (monomer 36 kDa), further supporting the idea that ASP1 exists as a trimer in solution.

**Figure 2:**
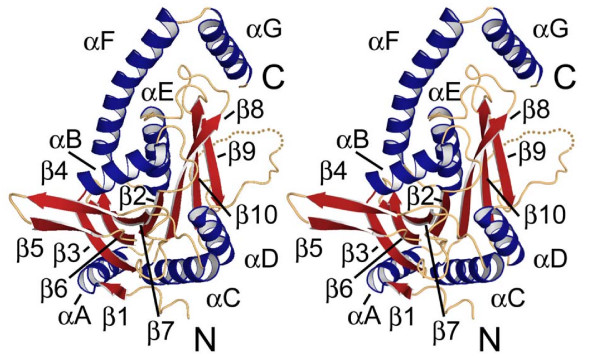
**Stereo image of ASP1 monomer structure**. β strands and α helices are in red and blue, respectively. A single disordered loop between β9-αD is shown as a dotted line.

**Figure 3:**
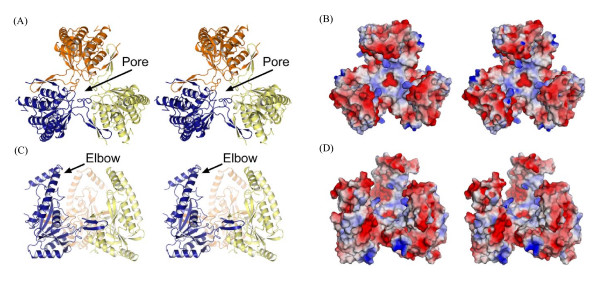
**Stereo image of ASP1 trimer**. (A) and (C) Orthoganol views of ASP1 trimer shown in ribbon. Individual subunits are colored, yellow, blue and orange. (B) and (D) Surface representations corresponding to views of ASP1 in (A) and (C), respectively. Positive and negative electrostatic potential are indicated in blue and red surface, respectively.

ASP1 contains a single domain comprised of a central mixed β-sheet (β1–3-6-7-2-8-10-9) flanked by 4 α-helices on one side and 3 on the other yielding an overall three layered αβα fold (Figure 2). Helices F and G form an elbow-shaped extension that is peripheral to the core domain. Based on secondary structure prediction of the missing 36 C-terminal residues, an additional or perhaps extended helix is expected to follow αG. Trimer formation is largely stabilized by interactions between an extended anti-parallel hairpin (β4–5) and the αD region from an adjacent monomer (Figure 2). A portion of the extended loop (residues 175–179) preceding αD further stabilizes the trimer through interactions with β4–5, resulting in formation of a 3-stranded anti-parallel sheet (Figure 3).

Assembly of the ASP1 trimer results in the formation of a roughly globular complex (~diameter 70 Ǻ) with three notable features (Figure 3). First, one side of the trimer adopts a very flat surface, forming what could perhaps function as a large docking interface. The electrostatic potential on this surface is quite neutral having only a small amount of basic potential. A second unusual feature of the ASP1 trimer is the presence of a large internal cavity (~7500 Ǻ^3^) surrounded by a three-pronged claw-like structure. Given the size of this cavity and overall claw-like structure that surrounds it, it is quite possible that this region acts as a binding surface for another protein(s) and or substrate. The electrostatic surface potential of each claw is negative creating an overall acidic surface on the internal cavity region of ASP1.

**Figure 4:**
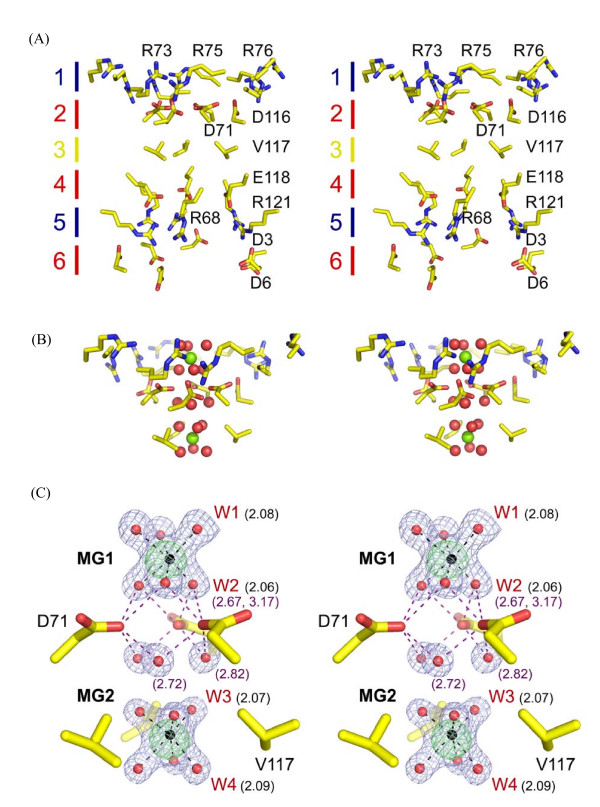
**Stereo image of central metal-binding pore**. (A) Amino acids lining the metal-binding pore are shown in stick representation. Concentric layers of amino acids are numbered and corresponding polarity indicated by color. Red, blue and yellow indicate negative, positive and neutral charge, respectively. (B) Interaction between upper 3 layers of central pore and hydrated Mg^2+ ^ions. Mg^2+ ^ions and surrounding water molecules are colored in green and red, respectively. (C) Magnesium coordination binding analysis. An Fo-Fc Mg^2+ ^omit map contoured at 5 σ is shown in green mesh. For reference, a 2Fo-Fc map contoured at 1.5 σ (blue mesh) is also shown for Mg^2+ ^ions (black sphere) and water molecules (red sphere) in the central pore region. Since the trimmeric structure presented here is generated using three-fold crystallographic symmetry only unique features are indicated. Water molecules bond to Mg^2+ ^ions are labeled in red W1 to W4. Distances in Ǻ are indicated in parenthesis with black and purple corresponding to water-metal and water-side chain distances, respectively. Interactions with both metals are indicated as black dashed bonds, while those involving D71 are shown in light purple.

The final and most notable feature of the ASP1 trimer is the presence of two well ordered magnesium ions (see Figure 4C for bonding distance and geometry) located at a central pore formed along the central three fold symmetry axis. This pore is ~20 Ǻ deep and is lined by six concentric rings of amino acids with alternating charge and polarity (Figure 3 and 4A). The shape of the pore is conical and is tapered to its narrowest point of 4.14 Ǻ at D71 located within the second layer. The first Mg^2+ ^ion is positioned just above a negative ring of amino acids formed by three copies of D71 and D116 (Figure 4). Water molecules in the first hydration shell of this metal ion are directly hydrogen bonded to D71 (Figure 4C). A second metal ion is located in a hydrophobic pocket lined by V117 at the third layer. Water molecules within the first hydration shell of this metal ion are in direct van der Waals contact with V117. Through its second hydration shell the second Mg^2+ ^is further stabilized by hydrogen bonding to D71 and also to the main chain carbonyl of R68. Because there was high concentration of Mg^2+ ^in the mother liquor (~0.55 M), the specificity and possible role of this metal ion-bound, channel-like pore is unclear, but may be involved in the biological function of the ASP1 trimer.

Although it is tempting to speculate that the presence of two Mg^2+ ^ions in the central pore region of ASP1 suggests a role for ASP1 in metal transport, there is no direct evidence to support this idea. Furthermore, a structural comparison of ASP1 with CorA, a well characterized Mg^2+ ^transporter whose homologs are present in *S. coelicolor *and various actinobacteria [23,24], shows no obvious similarity between these proteins (results not shown). Therefore, if ASP1 function does involve some aspect of Mg^2+ ^binding and/or transporter it does not appear to be similar to that conducted by CorA.

### Structural Comparisons of ASP1

To further characterize the structure of ASP1 and gain insight into its possible function, we performed a comparative structural analysis using the program DaliLite version 3 [25]. This analysis revealed significant structural similarity to a homologue from *Corynebacterium glutamicum *(GeneID: Ncgl1848) [PDB: 2P90], as well as several bacterial purine nucleoside phosphorylases and a number of other glycosidic hydrolases from the larger NP-1 family.

#### Comparison of ASP1 from S. coelicolor and C. glutamicum

As expected, structural comparison of ASP1 from *S. coelicolor *and *C. glutamicum *showed a high degree of conservation (root mean square deviation (RMSD): 1.6 Ǻ). Importantly, the structure of ASP1 from *C. glutamicum *crystallized as a trimer that is identical to the trimer reported here for ASP1 from *S. coelicolor*. This finding, along with our gel filtration data, provides additional support for the trimeric structure of ASP1 generated through crystallographic symmetry. Another important observation from the comparison of the structure from *C. glutamicum *is the structural conservation of the metal binding pore despite the absence of bound metal ion. The fact that the pore region adopts an identical structure even when a metal ion is not present provides strong evidence to suggest that the binding of metal is not simply required for structure integrity of the ASP1 trimer.

**Figure 5:**
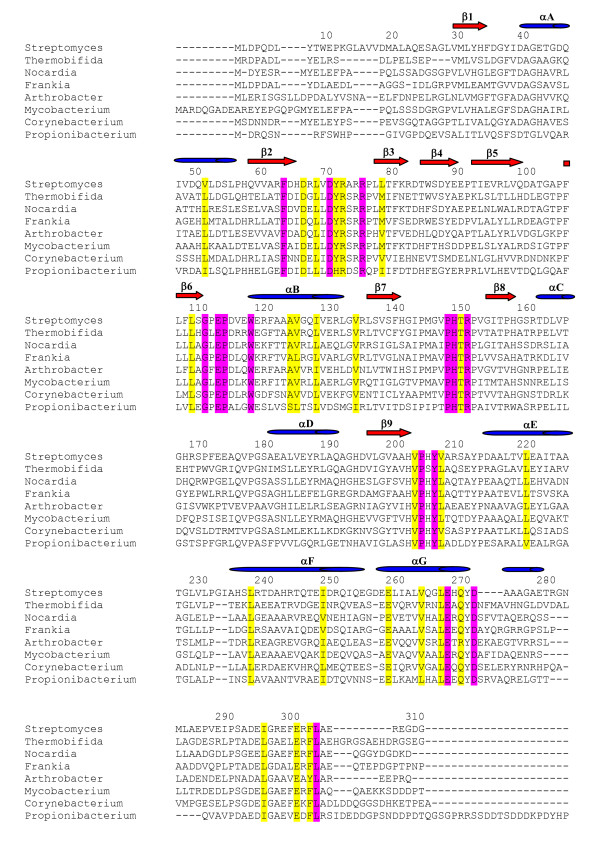
**Multiple sequence alignments of ASP1 homologues from 8 representative actinobacterial species**. Conserved residues are colored based on the complete alignment of all 43 available actinobacterial homologues (see Additional file 3): purple, absolutely conserved residues; yellow, highly conserved residues. β strands and α helices are labeled in red and blue, corresponding to the ribbon diagram in Figure 2.

**Figure 6:**
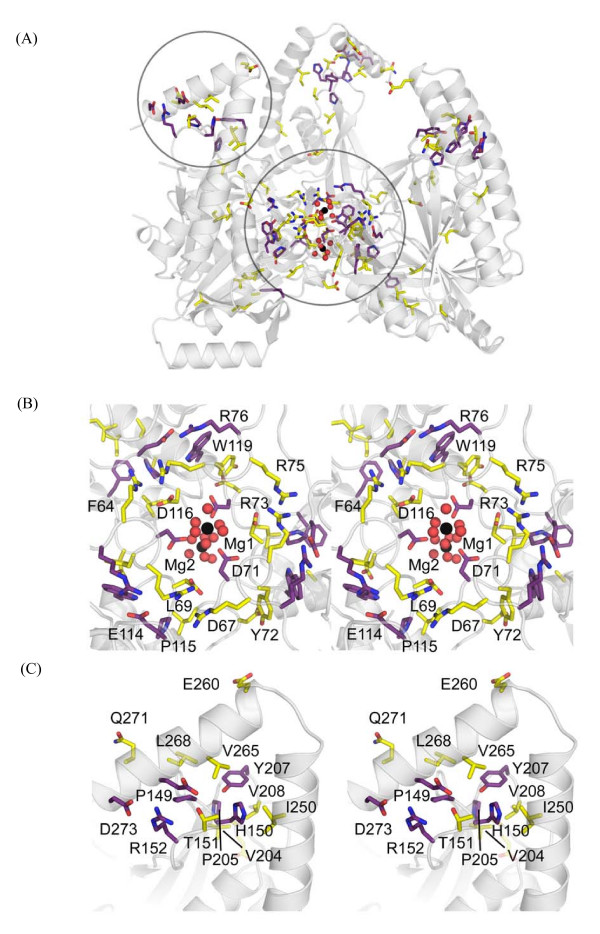
**Highly conserved regions within the ASP1 trimer**. (A) Conserved residues important for forming the central pore and elbow regions. A circle delineates each region. Absolutely conserved residues are colored in purple and highly conserved ones are colored in yellow. (B) and (C) Stereo views of the central pore region and C-terminal elbow regions, respectively. Conserved residues are indicated as in (A). Magnesium ions are shown as black spheres with their first hydration shell of water molecules shown as red spheres.

A comprehensive sequence alignment of ASP1 homologues from a broad range of actinobacterial species (Figure 5 and Additional file 3) clearly demonstrates that residues contributing to the formation of two distinct regions (the central pore and C-terminal elbow) within the structure of ASP1 represent the most highly conserved sequence of the protein (Figure 6). Figure 6 illustrates the importance of conserved residues (absolutely conserved in purple, highly conserved in yellow) in forming the pore and elbow regions. While most of these residues are involved in structural stabilization others, such as D71 and L268, are not. As suggested elsewhere, absolutely conserved amino acids that do not directly contribute to structure stability and are solvent exposed, are expected to define key regions for protein function [26-29]. At this point it is difficult to infer what function the elbow region might serve. Given its distal location, however, it seems likely to mediate interaction with other proteins or perhaps the missing C-terminal region of ASP1. The C-terminal region of ASP1 contains a number of highly conserved residues (I296, E302, F304, L305). Interestingly, this region is not observed in either of the currently available structures suggesting that it may only become ordered upon binding another molecule.

#### Comparison of ASP1 and PNP

**Figure 7:**
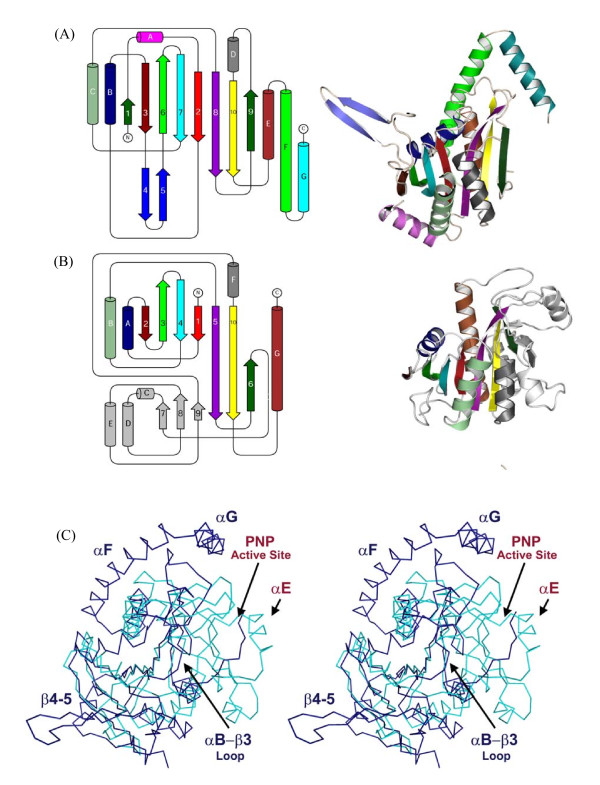
**Structural and Topological Comparison of (A) ASP1 and (B) PNP_*E. coli *_[PDB**: 1ECP]. (C) Structural Comparison of ASP1 monomer (dark blue) with PNP_*E. coli *_(light blue) [PDB: 1ECP]. Regions defining ASP1 structure are labeled blue, while those referring to PNP_*E. coli *_are labeled red.

As mentioned above, comparative structural analysis revealed significant similarity (Z score ~10) between ASP1 and members of the NP-1 family of nucleoside phosphorylase enzymes. This family of enzymes participates in the salvage pathway of purines and pyrimidines biosynthesis and catalyzes the reversible phosphorolysis of purine and pyrimidine nucleosides [18,19]. The NP-1 family member that shares greatest structural similarity to ASP1 is purine nucleoside phosphorylase (PNP) from *E. coli *[PDB: 1ECP]. Despite having very low sequence similarity (8% identity), ASP1 and PNP_*E. coli *_structures could be aligned with an overall RMSD of ~3.0 Ǻ. With the exception of a few insertions and deletions, these proteins share identical overall topology (Figure 7). Two notable insertions include: the C-terminal highly conserved elbow (αF-G) and the extended arm region (β4–5) essential for ASP1 trimer stability. In addition, there is an insertion of sequence that significantly increases the loop size between αB-β3, occluding much of the normal PNP substrate-binding surface (Figure 7C). While these insertions are expected to contribute to ASP1 function and certainly quaternary structure, members of the NP-1 family are characterized by different oligomeric arrangements ranging from dimer, to trimer and in some instances hexameric structures. Therefore, these observed differences do not necessarily preclude shared function between PNP and ASP1.

In contrast, the following evidence strongly suggests that ASP1 does not function as a nucleoside phosphorylase. First, a large region of PNP responsible for forming an entire side of its active site cleft (residues ~100–180 encompassing β7–8–9 and αC-D-E; Figure 7B) is completely missing in the ASP1 structure, rendering ASP1 incompatible of binding nucleoside. Second, a sequence alignment (Additional file 3) of ASP1 homologues fails to identify any of the highly conserved residues involved in substrate binding or catalysis within the NP-1 family. Furthermore, from sequence and structural alignments it is equally clear that those regions of ASP1 which are most highly conserved, are not present within NP-1 family members. Finally, a PNP homologue in the *S. coelicolor *genome has already been identified (SCO4917) and shows no significant similarity to ASP1. Taken together, the observations from both sequence and structural comparison indicate that while ASP1 and PNP share similar overall structure and topology, their functions are different.

### Phylogenetic Analysis of ASP1 and ASP2

Of the five actinobacteria-specific genes previously identified through comparative genomic analysis of 19 actinobacterial species, two genes ASP1 (SCO1997; ML1009) and ASP2 (SCO1662; ML1306) appear to encode structurally related proteins [1]. These proteins have comparable length and share significant sequence similarity (25% identity and 43% similarity). The question remains, are these two conserved actinobacteria-specific proteins functionally related?

**Figure 8:**
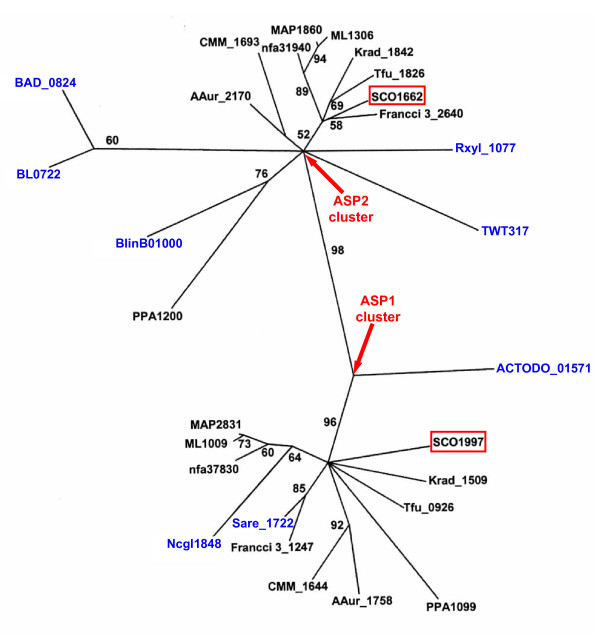
**Phylogenetic tree of ASP1 and ASP2 homologous genes in different actinobacterial species**. The gene ID in blue are from these genomes which only contain one of the two paralogous genes. The representing genomes of each gene ID are listed in Additional file 2. The bootstrap scores >50% are indicated on various branch points.

We have conducted a search for ASP1 and ASP2 homologues in all available sequenced actinobacterial genomes (61 strains). Interestingly, while most actinobacterial species contain homologues of both ASP1 and ASP2, some species contain only one homologue. The single homologue by definition shares similarity to both ASP1 and ASP2 (see Additional file 2). Species (18 in total), which only contain one homologue, are found in 7 divergent genera (*Corynebacterium, Actinomyces, Saccharopolyspora, Brevibacterium, Bifidobacterium, Tropheryma *and *Rubrobacter*). Further phylogenetic analysis of ASP1 and ASP2 homologues from different actinobacterial species was conducted to determine how these two genes are related. In the phylogenetic tree shown in Figure 8, two distinct clusters are observed with a strong bootstrap score (98%) indicating that the observed branch pattern is highly reliable. One cluster collected all genes homologous to ASP2 while the other cluster, grouped only those genes homologous to ASP1. The genes from the 18 species containing only one homologue do not form a third branch, but rather fall into either the ASP1 and ASP2 clusters. The two distinct clusters observed in the phylogenetic tree suggest that ASP1 and ASP2 are paralogues that evolved from a gene duplication event in a common ancestor of actinobacteria. Therefore, most members from this phylum contain both ASP1 and ASP2 except those species, which have lost one copy later in the evolutionary process. The fact that ASP1 and ASP2 are paralogues, yet either can be lost, suggests that these two paralogues perform similar functions. Based on their sequence and functional similarity, these two proteins are also expected to share significant structural similarity. Preliminary X-ray crystallographic analysis indicates that the tertiary and quaternary structure of ASP2 is in fact similar to ASP1 (data not shown).

Sequence alignment of ASP1 and ASP2 homologues demonstrate that important residues which are highly conserved in ASP1 homologues and likely involved in protein function are also conserved in ASP2 homologues (Additional file 4). As highlighted in Additional file 4, 8 of the 15 absolutely conserved residues from ASP1 homologues are also absolutely conserved amongst ASP2 homologues. The remaining 7 are still highly conserved and are only substituted with similar amino acids (Additional file 4). This finding further underscores the importance of these residues in mediating the function of both paralogs. As stated earlier, amino acids that fall within the category of absolutely conserved and solvent exposed are particularly predictive of regions important for mediating interactions with other functionally important molecules [26-29]. D71 is most interesting in this regard because it not only fits this category, but is also found bound to two magnesium ions in the ASP1 structure. We know that the binding of magnesium is not required for overall structural stability since the structure of ASP1 from *C. glutamicum *does not contain metal ion. The precise function of this region within ASP1 and ASP2 will require further investigation.

## Conclusion

The Actinobacteria phylum represents one of the largest groups of bacteria. Amazingly this diverse collection of bacteria can be characterized genetically to a first approximation by the presence of only 5 unique genes. All of these 5 genes, are of unknown function but they are expected to encode for function(s) that ultimately control actinobacteria-specific and important biological process(es). Understanding the cellular function of a protein of unknown function is not a straightforward task [20,30]. However, structure determination often provides the most useful information in this regard [20,30]. In this work, we report the structure of the first actinobacteria-specific protein. Our structural data in combination with sequence analysis further supports the idea that this protein carries out a novel function. This function is novel in the sense that the structure of this protein does not match any known protein, with or without known function. Given the immense number of structures that are now available and the wide coverage of function, it is reasonable to propose that ASP1 may mediate a function highly specific to Actinobacteria. Although it is unclear from the structural data alone, it seems possible that ASP1 function may involve some aspect of divalent metal ion interaction. It will be intriguing to determine what contribution, if any, this highly conserved 'pore' region makes toward ASP1 function. Our phylogenetic analysis also shows that another actinobacteria-specific protein ASP2, which is a paralogue of ASP1, may also have similar structure and function. Future genetic and biochemical studies of these proteins is therefore of great interest in linking the conservation of the biology of actinobacteria and their 5 unique genes.

## Methods

### Protein Expression and Purification

The ASP1 gene (SCO1997) from *S. coelicolor *A3(2) was cloned into the pET-22b vector and expressed in *E. coli *BL21(DE3) as a full length recombinant protein with a C-terminal (His)_6_-tag. SeMet protein was expressed in the methionine auxotroph *E. coli *B834 using a previously described method [31]. For expression of both native and SeMet derivatized ASP1, cells were grown at 37°C to an OD_600 _of ~0.6; induced with 1 mM isopropyl beta-D-thiogalactopyranoside (IPTG); harvested after 4 h; resuspended in a binding buffer containing 20 mM Tris, pH 7.4, 500 mM NaCl and 10 mM imidazole; lysed in a French pressure cell; and clarified by centrifugation. Supernatant was loaded on a 1 mL Ni-column, and washed with 200 mL binding buffer along with 36 mM imidazole, and finally eluted at 300 mM imidazole. The eluted proteins were diluted 5 fold with buffer A (20 mM Tris, pH 7.5) and loaded onto a 5 mL HiTrap Q HP anion exchange column (Amersham) for further purification. Proteins were eluted with a 120 mL linear gradient from 50 to 500 mM NaCl. ASP1 eluted as a single peak at ~260 mM NaCl. Individual fractions from across the peak were pooled and buffer exchanged into a low-salt buffer (25 mM KCl, 10 mM HEPES, pH 7.5) for crystallization. The buffer used for gel filtration chromatography contained 20 mM Tris (pH 7.4) and 200 mM KCl.

### Crystallization and Data Collection of ASP1

All crystals were grown at 17°C using the hanging drop/vapour diffusion method. Hanging drops containing 1 uL of protein solution (5 mg/mL) and 1 uL of mother liquor (0.1 M MES, 0.55 M magnesium formate, pH6.5~6.8, 0.25~0.5% n-Octyl-beta-D-glucoside, 0~1.5% glycerol) were dehydrated over a reservoir containing 800 uL of 1.5 M (NH_4_)_2_SO_4_. Cubic shaped crystals (100 × 100 × 100 μm^3^), suitable for data collection, grew after approximately 3 days incubation. Crystals were flash frozen directly in a nitrogen cold stream (100 K) with no further cryo-protection. Diffraction data sets for native and SeMet crystals were collected at wavelengths of 1.1 and 0.979 Å, respectively. All data was collected at the X25 beamline using an ADSC Q315 CCD x-ray detector (NSLS, Brookhaven, NY).

### Structure Determination and Model Refinement

SAD data collected to 2.0 Å was processed using d*TREK [32]. All 5 of the expected SeMet sites were located using *HYSS *[33,34]. Phasing and density modification were carried out using *CNS *[35]. Iterative rounds of manual model building and refinement were performed with Coot and *REFMAC5 *until R and R_free _values converged and could no longer be improved [36,37]. The coordinates of the final ASP1 model were deposited in the Protein Data Bank under accession code 3E35. Surface area calculations were performed using the program *PISA *version 1.15 [38]. Structure similarity searches were performed by *DaliLite *program v3 [25]. Structural illustrations presented in figures were generated with *PyMOL *[39].

### Phylogenetic Analysis

Phylogenetic analyses were carried out based on sequence alignments for ASP1 and ASP2 homologous genes from 18 actinobacterial species. Among these selected species, only 8 contain one of the two genes, while the others contain both gene copies. Multiple sequence alignments were created using the *ClustalX *version 1.83 [40]. The alignment was then imported into *TREE-PUZZLE *version 5.2 for maximum-likelihood (ML) analysis using the WAG+F model with gamma distribution of evolutionary rates with four categories [41,42].

## Authors' contributions

BG carried out the crystallization of ASP1 under MSJ's supervision, and performed phylogenetic analyses of ASP1. MSJ contributed in structural data collection and structure determination with the help of SSM. BG and MSJ conducted structural analyses and prepared the manuscript. RSG conceived and directed this study and was responsible for the final evaluation of the results. All authors have read and approved the manuscript.

## Supplementary Material

Additional file 1 **Gene ID and Possible Function of ASP1 homologues and its neighboring genes in all sequenced actinobacterial strains**. For the adjacent genes of ASP1 (two upstream and two downstream) in all sequenced actinobacterial strains, their possible function are indicated in brackets below their gene ID. *: incomplete genome. Click here for file

Additional file 2 **Gene ID and Accession no. of ASP1 and ASP2 homologues in all sequenced actinobacterial strains**. Click here for file

Additional file 3 **Alignment of all available ASP1 homologues from actinobacterial species**. Conserved residues are colored as such: purple, absolutely conserved residues; yellow, highly conserved. β strands and α helices are labeled in red and blue, corresponding to the ribbon diagram in Figure 2. Click here for file

Additional file 4 **Alignment of all available ASP1 and ASP2 homologues from actinobacterial species**. Conserved residues colored in Additional file 2 are also highlighted here. Unexpected amino acids at conserved positions are highlighted in cyan. Click here for file
