# Supplementary material for: Structural and phylogenetic analysis of a conserved actinobacteria-specific protein (ASP1; SCO1997) from Streptomyces coelicolor
Source: BMC Struct Biol. 2009 Jun 10;9:40. doi: 10.1186/1472-6807-9-40 (PMC2714318; doi:10.1186/1472-6807-9-40)
Supplement: Additional file 1 — Gene ID and Possible Function of ASP1 homologues and its neighboring genes in all sequenced actinobacterial strains. For the adjacent genes of ASP1 (two upstream and two downstream) in all sequenced actinobacterial strains, their possible function are indicated in brackets below their gene ID. *: incomplete genome. [file 1472-6807-9-40-S1.pdf]

Additional file 1: Gene ID and Possible Function of ASP1 homologues and its neighboring genes in all sequenced actinobacterial strains.

Actinobacterial strain	Left to ASP1		ASP1	Right to APS1	
Streptomyces coelicolor A3(2)	SCO1995<br>[Unkown]	SCO1996<br>[CoaE]	SCO1997<br>[Unkown]	SCO1998<br>[RpsA]	SCO1999<br>[SAM]
Streptomyces avermitilis MA-4680	SAV_6232<br>[SAM]	SAV_6233<br>[RpsA]	SAV_6234<br>[Unkown]	SAV_6235<br>[CoaE]	SAV_6236<br>[Unkown]
Streptomyces griseus subsp. Griseus NBRC 13350	SGR_5534<br>[RpsA]	SGR_5535<br>[Unkown]	SGR_5536<br>[Unkown]	SGR_5537<br>[CoaE]	SGR_5538<br>[Unkown]
Streptomyces sp. Mg1	SSAG_03956<br>[Unkown]	SSAG_03957<br>[CoaE]	SSAG_03958<br>[Unkown]	SSAG_03959<br>[RpsA]	SSAG_03960<br>[SAM]
Thermobifida fusca YX	Tfu_0924<br>[ADP_ribosyl_GH]	Tfu_0925<br>[P-loop]	Tfu_0926<br>[Unkown]	Tfu_0927<br>[PyrD]	Tfu_0928<br>[FRK]
Salinispora arenicola CNS-205	Sare_1720<br>[sulfotransferase]	Sare_1721<br>[exonuclease]	Sare_1722<br>[Unkown]	Sare_1723<br>[ABM]	Sare_1724<br>[Unkown]
Salinispora tropica CNB-440	Strop_1735<br>[sulfotransferase]	Strop_1736<br>[exonuclease]	Strop_1737<br>[Unkown]	Strop_1738<br>[Unkown]	Strop_1739<br>[GDSL]
Kineococcus radiotolerans SRS30216	Krad_1507<br>[LysM]	Krad_1508<br>[ATP-cone]	Krad_1509<br>[Unkown]	Krad_1510<br>[Unkown]	Krad_1511<br>[regulator]
Rhodococcus jostii RHA1	RHA1_ro06803<br>[helicase]	RHA1_ro06804<br>[PldB]	RHA1_ro06805<br>[Unkown]	RHA1_ro06806<br>[Lactamase]	RHA1_ro06807<br>[Unkown]
Nocardia farcinica IFM 10152	nfa37810<br>[Unkown]	nfa37820<br>[Unkown]	nfa37830<br>[Unkown]	nfa37840<br>[helicase]	nfa37850<br>[LysA]
Frankia sp. CcI3	Francci3_1245<br>[Unkown]	Francci3_1246<br>[Unkown]	Francci3_1247<br>[Unkown]	Francci3_1248<br>[Unkown]	Francci3_1249<br>[Mem_trans]
Frankia alni ACN14a	FRAAL1974<br>[HisKA]	FRAAL1975<br>[Unkown]	FRAAL1976<br>[Unkown]	FRAAL1977<br>[Unkown]	FRAAL1978<br>[DFR]
Frankia sp. EAN1pec	Franean1_5206<br>[deacetylase]	Franean1_5207<br>[Unkown]	Franean1_5208<br>[Unkown]	Franean1_5209<br>[PhaG]	Franean1_5210<br>[Unkown]
Acidothermus cellulolyticus 11B	Acel_0026<br>[ZntA]	Acel_0027<br>[Lipoprotein]	Acel_0028<br>[Unkown]	Acel_0029<br>[GlpA]	Acel_0030<br>[amidohydrolase]
Mycobacterium abscessus	MAB_3031c<br>[Unkown]	MAB_3032<br>[SthA]	MAB_3033<br>[Unkown]	MAB_3034<br>[PldB]	MAB_3035<br>[Unkown]
Mycobacterium sp. MCS	Mmcs_2171<br>[PhzC]	Mmcs_2172<br>[PldB]	Mmcs_2173<br>[Unkown]	Mmcs_2174<br>[Unkown]	Mmcs_2175<br>[Unkown]
Mycobacterium sp. KMS	Mkms_2217<br>[PhzF]	Mkms_2218<br>[PldB]	Mkms_2219<br>[Unkown]	Mkms_2220<br>[Unkown]	Mkms_2221<br>[Unkown]
Mycobacterium sp. JLS	Mjls_2160<br>[PhzF]	Mjls_2161<br>[PldB]	Mjls_2162<br>[Unkown]	Mjls_2163<br>[Unkown]	Mjls_2164<br>[Unkown]
Mycobacterium vanbaalenii PYR-1	Mvan_2444<br>[PhzF]	Mvan_2445<br>[PldB]	Mvan_2446<br>[Unkown]	Mvan_2447<br>[Unkown]	Mvan_2448<br>[SthA]
Mycobacterium avium subsp. Paratuberculosis	MAP2829<br>[SthA]	MAP2830c<br>[Unkown]	MAP2831<br>[Unkown]	MAP2832<br>[PldB]	MAP2833c<br>[Unkown]
Mycobacterium avium 104	MAV_3606<br>[SthA]	MAV_3607<br>[Unkown]	MAV_3608<br>[Unkown]	MAV_3609<br>[PldB]	MAV_3610<br>[PhzC]
Mycobacterium gilvum PYR-GCK	Mflv_3949<br>[FeoA]	Mflv_3950<br>[Unkown]	Mflv_3951<br>[Unkown]	Mflv_3952<br>[PldB]	Mflv_3953<br>[PhzF]
Mycobacterium leprae TN	ML1007<br>[Unkown]	ML1008<br>[Unkown]	ML1009<br>[Unkown]	ML1010<br>[Unkown]	ML1011<br>[Unkown]
Mycobacterium marinum M	MMAR_1996<br>[PhzC]	MMAR_1997<br>[PldB]	MMAR_1998<br>[Unkown]	MMAR_1999<br>[Unkown]	MMAR_2000<br>[SthA]
Mycobacterium smegmatis str. MC2 155	MSMEG_2744<br>[PhzC]	MSMEG_2745<br>[Unkown]	MSMEG_2746<br>[Unkown]	MSMEG_2747<br>[Unkown]	MSMEG_2748<br>[SthA]
Mycobacterium bovis AF2122/97	Mb2731c<br>[Unkown]	Mb2732<br>[SthA]	Mb2733<br>[Unkown]	Mb2734<br>[PldB]	Mb2735<br>[PhzC]
Mycobacterium bovis BCG str. Pasteur 1173P2	BCG_2725c<br>[Unkown]	BCG_2726<br>[SthA]	BCG_2727<br>[Unkown]	BCG_2728<br>[PldB]	BCG_2729<br>[PhzC]
Mycobacterium ulcerans Agy99	MUL_3355<br>[Transposase]	MUL_3356<br>[Unkown]	MUL_3357<br>[Unkown]	MUL_3358<br>[PldB]	MUL_3359<br>[PhzC]
Mycobacterium tuberculosis H37Rv	Rv2712c<br>[Unkown]	Rv2713<br>[SthA]	Rv2714<br>[Unkown]	Rv2715<br>[PldB]	Rv2716<br>[PhzC]
Mycobacterium tuberculosis H37Ra	MRA_2740<br>[Unkown]	MRA_2741<br>[SthA]	MRA_2742<br>[Unkown]	MRA_2743<br>[PldB]	MRA_2744<br>[PhzC]
Mycobacterium tuberculosis CDC1551	MT2785<br>[Unkown]	MT2786<br>[SthA]	MT2787<br>[Unkown]	MT2788<br>[PldB]	MT2789<br>[PhzC]
Nocardioides sp. JS614	Noca_2850<br>[TesB]	Noca_2851<br>[TesB]	Noca_2852<br>[Unkown]	Noca_2853<br>[thiolase]	Noca_2854<br>[FadJ]
Renibacterium salmoninarum ATCC 33209	RSal33209_2427<br>[Unkown]	RSal33209_2428<br>[MFS]	RSal33209_2429<br>[Unkown]	RSal33209_2430<br>[Pep]	RSal33209_2431<br>[SthA]
marine actinobacterium PHSC20C1*	N/A	N/A	A20C1_03668<br>[Unkown]	N/A	N/A
Janibacter sp. HTCC2649*	N/A	N/A	JNB_00545	N/A	N/A

			[Unkown]		
Arthrobacter aurescens TC1	AAur_1756<br>[SthA]	AAur_1757<br>[Pep]	AAur_1758<br>[Unkown]	AAur_1759<br>[MFS]	AAur_1760<br>[Unkown]
Arthrobacter sp. FB24	Arth_1612<br>[SthA]	Arth_1613<br>[Pep]	Arth_1614<br>[Unkown]	Arth_1615<br>[MFS]	Arth_1616<br>[Unkown]
Leifsonia xyli subsp. xyli str. CTCB07	Lxx10150<br>[SthA]	Lxx10160<br>[Pep]	Lxx10170<br>[Unkown]	Lxx10180<br>[MFS]	Lxx101902<br>[Sigma70]
Clavibacter michiganensis subsp. sepedonicus	CMS_1625<br>[SthA]	CMS_1626<br>[Pep]	CMS_1627<br>[Unkown]	CMS_1628<br>[MFS]	CMS_1629<br>[Sigma70]
Clavibacter michiganensis subsp. michiganensis	CMM_1642<br>[SthA]	CMM_1643<br>[Pep]	CMM_1644<br>[Unkown]	CMM_1645<br>[MFS]	CMM_1646<br>[Sigma70]
Micrococcus luteus NCTC 2665*	N/A	N/A	MlutDRAFT_2379<br>[Unkown]	N/A	N/A
Propionibacterium acnes KPA171202	PPA1097<br>[Unkown]	PPA1098<br>[DnaK]	PPA1099<br>[Unkown]	PPA1100<br>[cyclophilin]	PPA1101<br>[PldB]
Kocuria rhizophila DC2201	KRH_14370<br>[Unkown]	KRH_14380<br>[MFS]	KRH_14390<br>[Unkown]	KRH_14400<br>[Pep]	KRH_14410<br>[SthA]
Corynebacterium glutamicum ATCC 13032	NCgl1846<br>[GalE]	NCgl1847<br>[Unkown]	NCgl1848<br>[Unkown]	NCgl1849<br>[helicase]	NCgl1850<br>[OxyR]
Corynebacterium glutamicum R	cgR_1751<br>[Unkown]	cgR_1752v<br>[Unkown]	cgR_1753<br>[Unkown]	cgR_1754<br>[helicase]	cgR_1755<br>[OxyR]
Corynebacterium diphtheriae NCTC	DIP1415<br>[GalE]	DIP1416<br>[Unkown]	DIP1417<br>[Unkown]	DIP1418<br>[helicase]	DIP1419<br>[Unkown]
Corynebacterium urealyticum DSM 7109	cur_0888<br>[OxyR]	cur_0889<br>[helicase]	cur_0890<br>[Unkown]	cur_0891<br>[Unkown]	cur_0892<br>[GalE]
Corynebacterium jeikeium K411	jk1098<br>[GalE]	jk1099<br>[Unkown]	jk1100<br>[Unkown]	jk1101<br>[helicase]	jk1102<br>[OxyR]
Corynebacterium efficiens YS-314	CE1813<br>[GalE]	CE1814<br>[Unkown]	CE1815<br>[Unkown]	CE1816<br>[helicase]	CE1817<br>[OxyR]
Actinomyces odontolyticus ATCC 17982	ACTODO_01569<br>[Pol_IV_kappa]	ACTODO_01570<br>[Unkown]	ACTODO_01571<br>[Unkown]	ACTODO_01572<br>[helicase]	ACTODO_01573<br>[dehydrogenase]
Rubrobacter xylanophilus DSM 9941	Rxyl_1075<br>[asparaginase]	Rxyl_1076<br>[Aat]	Rxyl_1077<br>[Unkown]	Rxyl_1078<br>[Unkown]	Rxyl_1079<br>[PP-loop]
Saccharopolyspora erythraea NRRL 2338	SACE_2231<br>[deaminase]	SACE_2232<br>[chitinase]	SACE_2233<br>[Unkown]	SACE_2234<br>[Unkown]	SACE_2235<br>[HAD]
Brevibacterium linens BL2	BlinB01000023<br>[RecB]	BlinB01000024<br>[HAD]	BlinB01000025<br>[Unkown]	BlinB01000026<br>[CysRS]	BlinB01000027<br>[Unkown]
Bifidobacterium adolescentis L2-32*	BIFADO_01159<br>[Unkown]	BIFADO_01160<br>[BacA]	BIFADO_01161<br>[Unkown]	N/A	N/A
Bifidobacterium longum NCC2705	BL0720<br>[Unkown]	BL0721<br>[BacA]	BL0722<br>[Unkown]	BLt06<br>[tRNA-Gly]	BLt07<br>[tRNA-Cys]
Bifidobacterium adolescentis ATCC 15703	BAD_t0041<br>[tRNA-Ala]	BAD_t0042<br>[tRNA-Gln]	BAD_0824<br>[Unkown]	BAD_0825<br>[BacA]	BAD_0826<br>[Unkown]
Bifidobacterium longum DJO10A	BLD_0445<br>[FRK]	BLD_0446<br>[BacA]	BLD_0447<br>[Unkown]	BLD_t0006<br>[tRNA-Gly]	BLD_t0007<br>[tRNA-Cys]
Bifidobacterium animalis subsp. lactis HN019*	N/A	N/A	BIFLAC_03242<br>[Unkown]	N/A	N/A
Bifidobacterium dentium ATCC 27678	N/A	BIFDEN_02061<br>[Unkown]	BIFDEN_02062<br>[Unkown]	BIFDEN_02063<br>[BacA]	BIFDEN_02064<br>[FRK]
Tropheryma whipplei str. Twist	TWT315<br>[BacA]	TWT316<br>[CysRS]	TWT317<br>[Unkown]	TWT318<br>[SAM]	TWT319<br>[FKBP_C]
Tropheryma whipplei TW08/27	TW453<br>[FKBP_C]	TW454<br>[SAM]	TW455<br>[Unkown]	TW456<br>[CysRS]	TW457<br>[BacA]

Note: * : incomplete genome.

The possible function of each protein is indicated in brackets below the gene ID and abbreviations for protein functions are as follows: Aat: leucyl/phenylalanyl-tRNA--protein transferase; ABM: Antibiotic biosynthesis monooxygenase; ADP_ribosyl_GH: ADP-ribosylglycohydrolase; ATP-cone: ATP-cone domain-containing protein; BacA: Bacitracin resistance protein; CoaE: dephospho-CoA kinase; CysRS: Cysteinyl-tRNA synthetase; DnaK: Molecular chaperone; DFR: dihydroflavonol-4-reductase; FadJ: multifunctional fatty acid oxidation complex subunit alpha; FeoA: Iron dependent repressor; FKBP_C: FKBP-type peptidyl-prolyl cis-trans isomerase; FRK: fructokinase; GalE: UDP-glucose 4-epimerase; GDSL: GDSL family lipase; GlpA: Glycerol-3-phosphate dehydrogenase; HAD: Haloacid dehalogenase-like hydrolase; HisKA: Histidine kinase; HrdB: RNA polymerase sigma factor; LysA: Diaminopimelate decarboxylase; LysM: peptidoglycan-binding LysM; Mem_trans: Membrane transport protein; MFS: Major Facilitator Superfamily; OxyR: DNA-binding transcriptional regulator; Pep: Cytosol aminopeptidase family; PhaG: monovalent cation/proton antiporter, MnhG/PhaG subunit; PhzC/PhzF: phenazine biosynthesis protein; PldB: Lysophospholipase; P-loop: P-loop containing Nucleoside Triphosphate Hydrolases; Pol_IV_kappa: a member of the Y-family of DNA polymerases; PyrD: Dihydroorotate dehydrogenase; RpsA: 30S ribosomal protein S1; SAM: SAM-dependent methyltransferase; SthA: soluble pyridine nucleotide transhydrogenase; TesB: palmitoyl-CoA hydrolase; ZntA: Cation transport ATPase.
