# Supplementary material for: Structural and phylogenetic analysis of a conserved actinobacteria-specific protein (ASP1; SCO1997) from Streptomyces coelicolor
Source: BMC Struct Biol. 2009 Jun 10;9:40. doi: 10.1186/1472-6807-9-40 (PMC2714318; doi:10.1186/1472-6807-9-40)
Supplement: Additional file 2 — Gene ID and Accession no. of ASP1 and ASP2 homologues in all sequenced actinobacterial strains. [file 1472-6807-9-40-S2.pdf]

Additional file 2: Gene ID and Accession no. of ASP1 and ASP2 homologues in all sequenced actinobacterial strains.

Actinobacterial strain	ASP1	ASP2
Streptomyces coelicolor A3(2)	SCO1997 [GenBank: NP_626258]	SCO1662 [GenBank: NP_625937]
Streptomyces avermitilis MA-4680	SAV_6234 [GenBank: NP_827410]	SAV_6650 [GenBank: NP_827826]
Streptomyces griseus subsp. Griseus NBRC 13350	SGR_5536 [GenBank: YP_001827048]	SGR_5841[GenBank: YP_001827353]
Streptomyces sp. Mg1	SSAG_03958 [GenBank: YP_00218060]	SSAG_04705 [GenBank: YP_002179778]
Thermobifida fusca YX	Tfu_0926 [GenBank: YP_288987]	Tfu_1826 [GenBank: YP_289882]
Salinispora arenicola CNS-205	Sare_1722 [GenBank: YP_001536600]	Sare_2324 [GenBank: YP_001537173]
Salinispora tropica CNB-440	Strop_1737 [GenBank: YP_001158578]	Strop_2170 [GenBank: YP_001158999]
Kineococcus radiotolerans SRS30216	Krad_1509 [GenBank: YP_001361261]	Krad_1842 [GenBank: YP_001361592]
Rhodococcus jostii RHA1	RHA1_ro06805 [GenBank: YP_706735]	RHA1_ro00860 [GenBank: YP_700851]
Nocardia farcinica IFM 10152	nfa37830 [GenBank: YP_119995]	nfa31940 [GenBank: YP_119405]
Frankia sp. CcI3	Francci3_1247 [GenBank: YP_480354]	Francci3_2640 [GenBank: YP_481731]
Frankia alni ACN14a	FRAAL1976 [GenBank: YP_712208]	FRAAL2860 [GenBank: YP_713072]
Frankia sp. EAN1pec	Franean1_5208 [GenBank: YP_001509472]	Franean1_4890 [GenBank: YP_001509161]
Acidothermus cellulolyticus 11B	Acel_0028 [GenBank: YP_871790]	Acel_1173 [GenBank: YP_872931]
Mycobacterium abscessus	MAB_3033 [GenBank: YP_001703765]	MAB_2128c [GenBank: YP_001702863]
Mycobacterium sp. MCS	Mmcs_2173 [GenBank: YP_639337]	Mmcs_3150 [GenBank: YP_640313]
Mycobacterium sp. KMS	Mkms_2219 [GenBank: YP_938207]	Mkms_3212 [GenBank: YP_939196]
Mycobacterium sp. JLS	Mjls_2162 [GenBank: YP_001070439]	Mjls_3162 [GenBank: YP_001071432]
Mycobacterium vanbaalenii PYR-1	Mvan_2446 [GenBank: YP_953265]	Mvan_3475 [GenBank: YP_954276]
Mycobacterium avium subsp. Paratuberculosis K-10	MAP2831 [GenBank: NP_961765]	MAP1860 [GenBank: NP_960794]
Mycobacterium avium 104	MAV_3608 [GenBank: YP_882785]	MAV_2378 [GenBank: YP_881579]
Mycobacterium gilvum PYR-GCK	Mflv_3951 [GenBank: YP_001135210]	Mflv_3052 [GenBank: YP_001134317]
Mycobacterium leprae TN	ML1009 [GenBank: NP_301746]	ML1306 [GenBank: NP_301939]
Mycobacterium marinum M	MMAR_1998 [GenBank: YP_001850303]	MMAR_3107 [GenBank: YP_001851397]
Mycobacterium smegmatis str. MC2 155	MSMEG_2746 [GenBank: YP_887078]	MSMEG_4186 [GenBank: YP_888465.]
Mycobacterium bovis AF2122/97	Mb2733 [GenBank: NP_856379]	Mb2149 [GenBank: NP_855798]
Mycobacterium bovis BCG str. Pasteur 1173P2	BCG_2727 [GenBank: YP_978813]	BCG_2142 [GenBank: YP_978231]
Mycobacterium ulcerans Agy99	MUL_3357 [GenBank: YP_907010]	MUL_2357 [GenBank: YP_906192]
Mycobacterium tuberculosis H37Rv	Rv2714 [GenBank: NP_217230]	Rv2125 [GenBank: NP_216641]
Mycobacterium tuberculosis H37Ra	MRA_2742 [GenBank: YP_001284078]	MRA_2140 [GenBank: YP_001283463]
Mycobacterium tuberculosis CDC1551	MT2787 [GenBank: NP_337289]	MT2184 [GenBank: NP_336654]
Nocardioides sp. JS614	Noca_2852 [GenBank: YP_924041]	Noca_2630 [GenBank: YP_923820]
Renibacterium salmoninarum ATCC 33209	RSal33209_2429[GenBank:YP_001625569]	RSal33209_2043[GenBank:YP_001625190]
marine actinobacterium PHSC20C1	A20C1_03668 [GenBank: ZP_01131566]	A20C1_02494 [GenBank: ZP_01131235]
Janibacter sp. HTCC2649	JNB_00545 [GenBank: ZP_00994816]	JNB_03080 [GenBank: ZP_00995323]
Arthrobacter aurescens TC1	AAur_1758 [GenBank: YP_947518]	AAur_2170 [GenBank: YP_947912]
Arthrobacter sp. FB24	Arth_1614 [GenBank: YP_831108]	Arth_2169 [GenBank: YP_831649]
Leifsonia xyli subsp. xyli str. CTCB07	Lxx10170 [GenBank: YP_061989]	Lxx08270 [GenBank: YP_061833]
Clavibacter michiganensis subsp. sepedonicus	CMS_1627 [GenBank: YP_001710350]	CMS_1670 [GenBank: YP_001710391]
Clavibacter michiganensis subsp. michiganensis NCPPB 382	CMM_1644 [GenBank: YP_001222387]	CMM_1693 [GenBank: YP_001222436]
Micrococcus luteus NCTC 2665	MlutDRAFT_2379 [GenBank:ZP_02945884]	MlutDRAFT_0605 [GenBank:ZP_02944113]
Propionibacterium acnes KPA171202	PPA1099 [GenBank: YP_055804]	PPA1200 [GenBank: YP_055907]
Kocuria rhizophila DC2201	KRH_14390 [GenBank: YP_001855292]	KRH_13910 [GenBank: YP_001855244]
*Corynebacterium glutamicum ATCC 13032	NCgl1848 [GenBank: NP_601129]	
*Corynebacterium glutamicum R	cgR_1753 [GenBank: YP_001138649]	
*Corynebacterium diphtheriae NCTC	DIP1417 [GenBank: NP_939769]	
*Corynebacterium urealyticum DSM 7109	cur_0890 [GenBank: YP_001800284]	
*Corynebacterium jeikeium K411	jk1100 [GenBank: YP_250882]	
*Corynebacterium efficiens YS-314	CE1815 [GenBank: NP_738425]	
*Actinomyces odontolyticus ATCC 17982	ACTODO_01571 [GenBank: ZP_02044696]	
*Rubrobacter xylanophilus DSM 9941	Rxyl_1077 [GenBank: YP_643855]	
*Saccharopolyspora erythraea NRRL 2338	SACE_2233 [GenBank: YP_001104463]	
*Brevibacterium linens BL2	BlinB01000025 [GenBank: ZP_00381360]	
*Bifidobacterium adolescentis L2-32	BIFADO_01161 [GenBank: ZP_02028723]	
*Bifidobacterium longum NCC2705	BL0722 [GenBank: NP_695904]	
*Bifidobacterium adolescentis ATCC 15703	BAD_0824 [GenBank: YP_909687]	
*Bifidobacterium longum DJO10A	BLD_0447 [GenBank: YP_001954391]	
*Bifidobacterium animalis subsp. lactis HN019	BIFLAC_03242 [GenBank: ZP_02963004]	
*Bifidobacterium dentium ATCC 27678	BIFDEN_02062 [GenBank: ZP_02918747]	
*Tropheryma whipplei str. Twist	TWT317 [GenBank: NP_787445]	
*Tropheryma whipplei TW08/27	TW455 [GenBank: NP_789385]	

*These actinobacteria contain a single homologue for these two paralogous proteins.
