# Supplementary material for: Structural and phylogenetic analysis of a conserved actinobacteria-specific protein (ASP1; SCO1997) from Streptomyces coelicolor
Source: BMC Struct Biol. 2009 Jun 10;9:40. doi: 10.1186/1472-6807-9-40 (PMC2714318; doi:10.1186/1472-6807-9-40)
Supplement: Additional file 3 — Alignment of all available ASP1 homologues from actinobacterial species. Conserved residues are colored as such: purple, absolutely conserved residues; yellow, highly conserved. β strands and α helices are labeled in red and blue, corresponding to the ribbon diagram in Figure 2. [file 1472-6807-9-40-S3.pdf]

Additional file 3. Alignment of all available ASP1 homologues from actinobacterial species.

```
                                                                                                            β1              αA          β2
AAur_1758         MPSDVMSPGVMCWVPEPPRTLSLRKEHAVFERISGSLLDPES---LYARNIETFH---SP--------------ELRGLNMVMGFT---------GFADAGHVVRQINAELLDELGAEVV
CMM_1644          ------------------------------------MADADG---LYEIDTDIGE---VP----------------TGLPLVAGLT---------GFSDAGSGVSQVSEYLLSTLSHRDV
Acel_0028         ------------------------------------MLDPTD---LYTISGTPPAR--AP--------------DAPL-ILVHALS---------GYVDAGDAVRLTARHLLDTLDHREI
A20C1_03668       ------------------------------------MRDPAG---LYDIATDVAG---VP----------------RGLPLVAGLT---------GFADAGGAVSQFSQYLLDTLERSVV
Lxx10170          ------------------------------------MRDPGE---LFELTS-ALE---VP----------------DGLPLVAGLT---------GFADAGSGVSQLGTYLLATLHSEVV
MSMEG_2746        ------------------------MADN-ENPQQRYQPDQSG---MYELEFPAPQLSSSD--------------GRGP-VLIHALE---------GFSDAGHAIRLAAEHLKKSLDTELV
Mvan_2446         ------------------------MADDVHDPGHEYQPEQTG---MYELEFPGPQLSSPD--------------GRGP-VLIHALE---------GFSDAGHAIRLSAQHLKDTLDTELV
Mb2733            -----------------------MARDQGADEAREYEPGQPG---MYELEFPAPQLSSSD--------------GRGP-VLVHALE---------GFSDAGHAIRLAAAHLKAALDTELV
SGR_5536          ------------------------------------MPDPQS---LYEWEPKGL--------------------AVVDMALAQESAGLVMLYHFDGYIDAGETGEQIVDGLLETLPHQVV
ML1009            ---------------------MSHSHYQDPDDEQHYQPGQPG---MYVLEFPAPQLLASD--------------GRGP-VLIHALE---------GFSDAGHAIRLAATHLKAALNTELV
MAB_3033          ------------------------MTSNEGVEPQPYKPDQSG---MYELEFPGPQLASAD--------------GRGP-VLVHALQ---------GFSDSGHAVKLAAAHLRQTLESELV
SCO1997           ------------------------------------MLDPQD---LYTWEPKGL--------------------AVVDMALAQESAGLVMLYHFDGYIDAGETGDQIVDQVLDSLPHQVV
Strop_1737        ------------------------------------MLDPHE---LYELTDDLP--------------------DLGQPVLIQALS---------GFVDAGNATRLAREQLLTSLDARPV
Francci3_1247     ------------------------------------MLDPDA---LYDLAEDLAGG--SI--------------DLGRPVMLEAMT---------GVVDAGSAVSLAGEHLMTALDHRLL
JNB_00545         ------------------------------------MQNPSD---LYRFEFDGPA---PT--------------R-EPGALIVLLG---------AFIDAGNIQRILGTHLVDTSEAEVV
Noca_2852         ------------------------------------MPER-----LVHIVDEVPELDDAR--------------SRGALTMVLVLD---------GFLDAGNAAGRAAQHLVDLSEGPVV
MAP2831           -----------------------MTHRQDAGD--QYQPGQAG---MYELELPAPQLSTSD--------------GRGP-VLVHALE---------GFSDAGHAIRLAAKHLKAALDSELV
Rv2714            -----------------------MARDQGADEAREYEPGQPG---MYELEFPAPQLSSSD--------------GRGP-VLVHALE---------GFSDAGHAIRLAAAHLKAALDTELV
FRAAL1976         ------------------------------------MLDPQE---LYSLTEDASDD--PI--------------DAHRPIMLEALT---------GVVDAGNAVSLAGEHLLTALDHRIV
NCgl1848          ------------------------------------MSDNND--RMYELEYPSPEVSGQT---------------AGGPTLIVALQ---------GYADAGHAVESSSSHLMDALDHRLI
CE1815            ------------------------------------MSDNKE--SMYELEYPSPEVNGGG---------------SEGPTLIVALQ---------GYADAGHAVEASASHLMAALDHRLI
DIP1417           ------------------------------------MNEREA--KMYELEFPAPDVASDG---------------VDGPTLIIAMQ---------GYADAGLAVEASADHLLSALEHRPV
cu0890            ----------------------MPQ------DNSFGRRPRNA---MYEMEYPVPLEADSP---------------DEGIPMLIALQ---------GYADAGQAIAVSGNHLLEALESSVV
Mmcs_2173         ------------------------MADHPDIPEQQYQPEQTG---MYELEFPAPQLSAAD--------------GRGP-VLLHALE---------GFSDAGHVVRLATAHLKNSLDTELV
RHA1_ro06805      ------------------------------------MDEQSK---MYELEFPAPQLSSAD--------------GQGP-VLIHGLE---------GFSDAGHAVKLATTHLRESLESELV
nfa37830          ------------------------------------MDYESR---MYELEFPAPQLSSAD--------------GSGP-VLVHGLE---------GFTDAGHAVRLATTHLRESLESELV
Mflv_3951         ------------------------MADDAHDSGSRYQPEQTG---MYELEFPGPQLSTPD--------------GRGP-VLIHALE---------GFSDAGHAIKLAAAHLKNSLDTELV
MUL_3357          -----------------------MAHDQDPGEAQDYQPGQSG---MYELEFPAPQLSTSD--------------GRGP-VLVHALE---------GFSDAGHAIRLAATHLKDGLDTELV
Franean1_5208     ------------------------------------MLDPRE---LYEIRSDLD--------------------DLGRPVLIEAMT---------GVVDAGGAVGLASEHLTTALQHERI
MMAR_1998         -----------------------MAHDQDPGEAQDYQPGQSG---MYELEFPAPQLSTSD--------------GRGP-VLVHALE---------GFSDAGHAIRLAATHLKDGLDTELV
jk1100            ----------------------MHQHFENNGDEHNGRHERKAAGRMYEMEYPAPIQFDRPSIADEATGLGSAVEGSEELPMLVALQ---------GYADAGQAVSSASSHLLAALEHSPV
Sare_1722         ------------------------------------MLDPHE---LYELADDLP--------------------DLGQPVLIQALS---------GFVDAGNATRLAREQLLTSLDARPV
CMS_1627          -------------------------------------------------------------------------------------------------------MSQVSEYLLSTLSHRDV
SSAG_03958        ------------------------------------MLDPQG---LYEWDAKGL--------------------AVADLALAQDSAGLVMLYHFEGYIDAGEAGEQIVERLLDTLPHQLV
Tfu_0926          ------------------------------------MRDPAD---LYELRSDLP--------------------ELSEPVMLVSLD---------GFVDAGAAGKQAVATLLDGLQHTEL
SAV_6234          ------------------------------------MLDPQG---LYAWEPKGL--------------------AVVDMALAQESAGLVMLYHFDGYIDAGETGDQIVDRLLDSLPHQVV
Arth_1614         ----------------------------MLERISGSLLDPDA---LYVSNAELFD---NP--------------ELRGLNLVMGFT---------GFADAGHVVKQITAELLDTLESEVV
PPA1099           ------------------------------------MDRQSN---RFSWHPGIVG---PD--------------Q-EVSALITLVQ---------SFSDTGLVQARVRDAILSQLPHHEL
KRH_14390         ------------------------------------MLDPTA---LYLGNPALLG---DP--------------RLRGLPLVVALS---------GYVEAGHLAAQIEAVVLDSMPSETV
Krad_1509         ------------------------------------MLDPGS---LYSYEGERP--------------------ALVEPPLVVALS---------GFIDAGNAVRLSVEHLLATCRHEVV
RSal33209_2429    ---------------MTDRCGRIR----MKEIKYVPLRDPDT---LYATNGAMLQ---DE--------------GLRGLNLLMGFT---------GFSDAGQVVSQINAELLDKLDAEPV
ACTODO_01571      -----------------------------------MSINATD---LYADGPAAG---------------------AKAKILLIHFD---------GAIDAGAAGRMAIGQLLRSLHNERV
Rxyl_1077         -------------------------------------MDERYLDLEWRPELERP-------------------------VLVSAFT---------GWNDAAEAASMALSALGNAWGVRRF
                                                                                                                              :        .
```

```
                     β2               β3          β4        β5         β6               αB            β7               β8     αC
AAur_1758           AMFDADQLIDYRSRRPHISFV-----EDHLQDYQAPKLGLYKLNDGLGQPFLLLAGFEPDLQWERFSRAVVGIVEELDVNLVTWIHSIPMPVPHTRPVGVTVHGNMPDLIE-GISSWKPT
CMM_1644            LRFDTDTLLDYRARRPTIYFD-----QDHLADYRPARLALYLAHDEIGQPFLLLTGFEPDFRWEAFTAAVLGIVDRYRVSTTTWVHAIPMPVPHTRDINVTVSGNRTELID-ALSVWKPH
Acel_0028           ARFDIDQLFDYRARRPPMLFD-----EDHWAAYDAPELVVHAVTDGGGREFLLLTGPEPDTQWERFVAAVCTLLERYGVDVTVGLNAIPLATPHTRPIGVTRHATSQDLIP-GNRPWLQQ
A20C1_03668         ASFDNDALLDYRARRPTIYFD-----QDHLSDYRPAKLEVYLAKDELGQPFLLLTGYEPDFQWERFSAAVLRLVEKFDVKSTTWVHAIPMPVPHTRSIGVTVSGNRSELID-SLSVWKPH
Lxx10170            ATFDADILLDYRARRPIIYFD-----QDHLDGYQPSTLKLYLVYDELRQPFLLLSGFEPDFRWEAFTEAVLGLMERFRVKSVTWVHAIPMPVPHTRPIGVTVSGNRAELIE-AMSIWKPQ
MSMEG_2746          ASFAIDELLDYRSRRPLMTFK-----TDHFTAYEEPELNLYALHDTVGTPFLLLAGLEPDLRWERFITAVRLLAEQLGVRQVIGLGTIPMAVPHTRPVNLTAHSNNKELIA-EHTPWVGE
Mvan_2446           ASFAIDELLDYRSRRPLMTFK-----TDHFTHYDQPELNLYALRDTAGTPFLLLAGLEPDLRWERFITAVRLLSERLGVRRVIGLGSIPMAVPHTRPMTLTAHSNDKELIA-EHQPWVNE
Mb2733              ASFAIDELLDYRSRRPLMTFK-----TDHFTHSDDPELSLYALRDSIGTPFLLLAGLEPDLKWERFITAVRLLAERLGVRQTIGLGTVPMAVPHTRPITMTAHSNNRELIS-DFQPWISE
SGR_5536            ARFDHDRLVDYRARRPLLTFR-----RDRWTSFEAPALEVRVVQDATGAPFLLLSGPEPDVEWERFAAAVEQIVERLGVRLAVNFHGIPMGVPHTRPVGITPHGNRTDLMP-GHRSPFDE
ML1009              ASFAIDELLDYRSRRPLMTFK-----TDHFTHYDDPELSLYALRDSVGTPFLLLAGMEPDLKWERFITAVRLLAERLGVRQTISLGTVPMAVPHTRPITLTAHSNNGELIA-DFTPWITE
MAB_3033            ASFAIDDLLDYRSRRPVMTFK-----SDHFTEYATPELNLYALKDTKGTPFLLLAGLEPDLKWERFVNAIRLLAEQLGVRKTIGLGAIPMAVPHTRPITLTAHGNDRKTLD-EHPGWIDE
SCO1997             ARFDHDRLVDYRARRPLLTFK-----RDTWSDYEEPTIEVRLVQDATGAPFLFLSGPEPDVEWERFAAAVGQIVERLGVRLSVSFHGIPMGVPHTRPVGITPHGSRTDLVP-GHRSPFEE
Strop_1737          ARFDLDQLFDYRSRRPVMTFV-----EDHWESYDAPALELHLLRDDADTPFLLLTGPEPDLQWERFVAAVAGLSARLDVRLTVGLNAIPMAVPHTRRTGVTAHATRRELTA-GYEPWLQR
Francci3_1247       ATFDIDQLLDYRSRRPTMVFS-----EDRWESYEDPVLALYLLRDEAGTPFLLLAGPEPDLQWKRFTVALRGLVARLGVRLTVGLNAIPMAVPHTRPLVVSAHATRKDLIV-GYEPWLRR
JNB_00545           ATFDVDALLDYRGRRPAMVFD-----TNRWESYADPSMVLYRMSDRDGQTYYLLTGPEPDFQWERTIEAIRQLIQLLGISLVVTSHGIPMGVPHTRPVGMTAHATDPRLVG-GKESPFGQ
Noca_2852           ATFDVDEFHDYRARRPPMSFV-----RDHYDAYDAPRLVVRLLADTGGTPYLLLHGPEPDNRWEAFCRAVREVVERFGVSRVVGMGSVPMAVPHTRPIAITHHANSPELIT-GESPWRGE
MAP2831             ASFAIDELLDYRSRRPLMTFK-----TDHFTHYDDPELSLYALRDSVGTPFLLLAGLEPDLKWERFITAVRLLAERLGVRQTIGLGTVPMAVPHTRPITMTAHSNNPELIK-NFQPWIAE
Rv2714              ASFAIDELLDYRSRRPLMTFK-----TDHFTHSDDPELSLYALRDSIGTPFLLLAGLEPDLKWERFITAVRLLAERLGVRQTIGLGTVPMAVPHTRPITMTAHSNNRELIS-DFQPSISE
FRAAL1976           ATFDVDQLLDYRSRRPTMIFS-----EDHWESYTDPVLALYQLRDESDTPFLLLTGPEPDLQWKRFTAAVRGLVARLGVRLTVGLNAVPMAVPHTRPATITAHGSSKELVV-GYEPWLRR
NCgl1848            ASFNNDELIDYRSRRPVVVIE-----HNEVTSMDELNLGLHVVRDNDNKPFLMLSGPEPDLRWGDFSNAVVDLVEKFGVENTICLYAAPMTVPHTRPTVVTAHGNSTDRLK-DQVSLDTR
CE1815              ASFNNDELIDYRSRRPTVVID-----NNEVTEMDELQLGLHVVRDSDNKPFLVLSGPEPDLRWGAFTEAVSDLVDKFGVDQTICLYAAPMTVPHTRPTVVSAHGNCAELLK-SRFSIDAR
DIP1417             AVFNNDELIDYRSRRPAVTID-----HNIVVDANELKLSMDVLRDSNGTPFLLLSGPEPDLRWDAFSQAVADLVRRFGVRRTICIYAAPMTVPHTRPLIVSAHGNSLDLIK-HHFSIDSK
cu0890              ASFNLDDLLDYRARRPSITID-----ENRITKSDKLSLDVRIVKDVNDRPFLLVAGPEPDLKWEAFGHAIVELAQRANVSRAISFYSAPMTVPHTRPLVVSAHASEPSLVR-DYHTWDAR
Mmcs_2173           ASFAIDELLDYRSRRPLMTFK-----TDHFSAYEEPELNLYAMHDTVGTPFLLLAGMEPDLRWERFITAVRLLAEQLGVRQTIGLGSIPMAVPHTRPVTMTAHSNNKELIA-EHTPWVGE
RHA1_ro06805        ASFAVDELVDYRSRRPTMTFK-----ADHFSDYDQPELNLYALKDTAGTPFLLLAGMEPDLRWERFTTAVRLLAEQLGVRRTVGINAIPMAIPHTRPLGVTAHSTNKDLIK-DHQRWSGE
nfa37830            ASFDVDELLDYRSRRPLMTFK-----TDHFSDYAEPELNLWALRDTAGTPFLLLAGLEPDLRWEKFTTAVRLLAEQLGVRRSIGLSAIPMAIPHTRPLGITAHSSDRSLIA-DHQRWPGE
Mflv_3951           ASFAIDELLDYRSRRPLMTFK-----TDHFTHYDEPELNLYALHDSVGTPFLLLSGMEPDLRWERFVTAIRLLAERLGVRRVIGLGSIPMAVPHTRPMTLTAHSNDKELIA-EHQPWVGE
MUL_3357            ASFAIDELLDYRSRRSMMTFK-----TDHFTKYDDPELSLYALRDSVGTPFLLLAGMEPDLKWERFITAVRLLAERLGVRQTIGLGTVPMAVPHTRPVTMTAHSNNPELIA-DFQPWISE
Franean1_5208       VTFDVDQLMDYRSRRPPMVFY-----EDHWESYDDPVLAIELLHDEAGTPFLLLCGPEPDLHWKRFTKAVQAVMAELGVRMSVGLNAIPMAVPHTRPCGVTAHATRKELLV-GYEPWVRR
MMAR_1998           ASFAIDELLDYRSRRPMMTFK-----TDHFTKYDDPELSLYALRDSVGTPFLLLAGMEPDLKWERFITAVRLLAERLGVRQTIGLGTVPMAVPHTRPVTMTAHSNNPELIA-DFQPWISE
jk1100              ASFKVDELIDYRSRRPGVTID-----NSKVMDSDDVRLDLHLLKDTQGRPFLLLSGLEPDLKWGAFSDAVVDLARRGQVGMVASLYSAPMTVPHTRPLVISAHSSDSSLIQ-DFHTWDSR
Sare_1722           ARFDLDQLFDYRSRRPVMTFV-----EDHWESYDAPALELHLLRDDADTPFLLLTGPEPDLQWERFVAAVAGLATRLDVRLTVGLNAIPMAVPHTRRTGVTAHATRRELTA-GYEPWLQR
CMS_1627            LRFDTDTLLDYRARRPTIYFD-----QDHLADYRPARLALYLAHDEIGQPFLLLTGFEPDFRWEAFTAAVLGIVDRYRVSTTTWVHAIPMPVPHTRDINVTVSGNRTELID-ALSVWKPH
SSAG_03958          ASFDADRLVDYRARRPLLTFQ-----RDHWSEFEEPRLEVRLVQDATGAPFLLLSGPEPDVEWERFAVAVRQIVERLGVRLSVNFHGIPMGVPHTRPVGITPHGNRTDLMP-GHRSPFDE
Tfu_0926            ATFDIDGLLDYRSRRPVMIFN-----ETTWVSYAEPKLSLTLLHDLEGTPFLLLHGLEPDRRWEGFTAAVRQLVERLSVRLTVCFYGIPMAVPHTRPVTATPHATRPELVT-EHTPWVGR
SAV_6234            ARFDHDRLVDYRARRPLLTFK-----RDRWTEYEEPTLDVRLVQDTTGAPFLLLSGPEPDVEWERFAAAVQQIVERLGVRLSVNFHGIPMGVPHTRPVGLTPHGNRNDLVP-GHRSPFEE
Arth_1614           AVFDADQLIDYRSRRPHVTFV-----EDHLQDYQAPTLALYRLVDGLGKPFLFLAGFEPDLQWERFARAVVRIVEHLDVNLVTWIHSIPMPVPHTRPVGVTVHGNRPELIE-GISVWKPT
PPA1099             GEFDIDLLLDHRDSRQPIIFD-----TDHFEGYERPRLVLHEVTDQLGQAFLVLEGPEPALGWESLVSSLTSLVDSMGIRLTVITDSIPIPTPHTRPAIVTRWASRPELIL-GSTSPFGR
KRH_14390           ARFDIDQLYDYRSRRPRVTFA-----QDHFEDWEAPELSLHVVTDGLDRHFLMLTGPEPDTQWERFASAVVGLAQRLQVSLVAVVGGIPMPVPHTRPVLVTAHGPRADLLP-RINGNRPT
Krad_1509           ATFDVDQLHDYRARRPTMTFG-----GQDWQDYEPPRLQVHRVHDEDGSSLLLLTGPEPDVQWERFTAAVWQLADELGVGATIVLSAAPTAVPHTRPSGVTASASRPELLD-GYRTWDVE
RSal33209_2429      AIFDIDQLIDYRSRRPQISFV-----EDHLTDYRQPEMVLYRMVDGLGQPFLFLAGNEPDLQWERFSAAVVALVERLDVNLVAWVHSIPMPVPHTRPIGVTVHGNRPDLIE-GMSSWKAT
ACTODO_01571        ATFDADALMDYRSHRPIVTVDN----WASSPDMVMPETVLDLVEDDMGNPILVLHGAEPDAHWESFTNAINELCERAGVEITFSLHGVPSGVPHTRPTPVHVQATDASLLPPGSGAISNH
Rxyl_1077           GRFDPEEFFDYQSTRPQVKLVDGVTRKIEWPENALYATAAGGVEALGGRGAVLLSGPEPNLRWRTFSETVVGLARELGVEMVVTLGALLADVPHSRPVPVAANAQEASLVE-DLGLSASR
                      *  : : *::  *  : .                                .: * **   *     ::  :     :             **:*        .
```

```
                          αD         β9               αE                   αF                    αG
AAur_1758         VDVPAAIGHILELRLTEAERNVAGYVIHVPHYLSEAEYPPAAVAGLEYLGAATSLMLP--TDRLREAGREVGRQIAEQIEA-SEEVQAVVTNLETRYD---EKSEGIVRRSLLAD-EN-D
CMM_1644          TQVPANALHLVEHRLHDAGHPVAGFVLLVPHYLADTEFPLAAVAALESISAATGLIFP--TDRLREEGRDFVGRIDEQVAG-NQELARLVTTLEERYD---SYMEDTPLKSPLTD-ED-G
Acel_0028         VHVPASAAHLLEFRLGEQGRKAMGIAVHVPHYVAQSEFPPAALRLIDELRERTGLDLP--REPIATAAAATLAEIDRQVGE-STDAMAMVSTLEQQYD---AFLHARSGESPLGGG-T-D
A20C1_03668       TQVPANAMHLIEYRLQELGHPTAGFVLLIPHYLADTEWPTAAVTALESVSAATGLIFP--TDRLREDGREFVAKIDSQVES-NQELARLVGTLEERHD---NYMEENPMRSPLTD-ED-G
Lxx10170          TQVPANALHLLEFRLQPLSYPIAGFVLLIPHYLADAEYPAAAITGLESISAATGLIFP--TDRLREDDRQFVANINEQVAG-NAELGRLVGTLENRHD---SYMEDNQPRSPLTD-RD-G
MSMEG_2746        VQVPASVSNLLEFRMAQHGHEVVGFTVHVPHYLAQTAYPPAAEALLAEVARTGSLELP--LAALSEAGAEVYTKINEQVEA-SPEVAQVVSALERQYD---AFIAAQENRS-LLARD--E
Mvan_2446         VQVPGSASNLLEFRMAQHGYEVVGFTVHVPHYLAQTDYPSAAETLLSEVARNGSLEIP--TTKLTQAAAEVFDKINEQVAG-SAEVAQVVEALERQYD---AFVAAQENRS-LLARD--E
Mb2733            IQVPGSASNLLEYRMAQHGHEVVGFTVHVPHYLTQTDYPAAAQALLEQVAKTGSLQLP--LAALAEAAAEVQAKIDEQVQA-SAEVAQVVAALERQYD---AFIDAQENRS-LLTRD--E
SGR_5536          AQVPGSAEALVEYRLMESGHDVLGVATHVPHYVARSAYPDAALTALEAITAATGLVLPALAHTLRTEAHRTQTEIDRQIGQGDEELVSLVEGLEHQYD---AVAGSETRGNLVAEP---V
ML1009            IQVPGSASNLLEYRMGQHGHEVVGFTVHVPHYLTQTDYPAAAQALLEQVAKTGALQLP--LSALAEAAAEIRAKIDEQVQA-STEVAQVVAALERQYD---AFIDAQENRS-LLRRD--E
MAB_3033          VQVPGSASNLLEFRLAQHGHDVVGFAVHVPHYLAQTDYPEASQRLLEEVARTGDLDLP--LQELSEAAAKVRNQINEQVEG-SEEVAQVVQALERQYD---AFVAAQENRS-LLARD--E
SCO1997           AQVPGSAEALVEYRLAQAGHDVLGVAAHVPHYVARSAYPDAALTVLEAITAATGLVLPGIAHSLRTDAHRTQTEIDRQIQEGDEELIALVQGLEHQYD---AAAGAETRGNMLAEP---V
Strop_1737        VQVPGSIGHLLEYRLGEQGRDALGFAAHVPHYVAQTEYPAAAEVLLASVSRSTGLLLP--SDGLRAATEAVRTEIDRQVAQ-TEDAAALVQALEEQYD---AFTRGRGQPNLLSTG-T-E
Francci3_1247     LQVPGSAGHLLEFELGREGRDAMGFAAHVPHYLAQTTYPAATEVLLTSVSKATGLLLP--LDGLRSAAVAIQDEVDSQIAR-GGEAAALVSALEEQYD---AYQRGRRGPSLPAADDV-Q
JNB_00545         IQVPASFPALLELRLGEAGRDALGFAIHVPQYLAQAEWAEGALAALNAITDATGLNLP--NDDLIAKAGINNREIASELND-NADAGQIVSALEQQYD---AFVEGQERPS-LLATEA-E
Noca_2852         LRIPSSAQALLEVRLGEWGHDAMGFVAHIPHYLAQMDYPRASAALLEQVEIAGRLTVD--LSGLRAEAEDREAEIARYLAA-NEEVAEVVAALERQYD---AFERAEESGTSLLARD--Q
MAP2831           IQVPGSASNLLEYRMAQHGHEVVGYTVHVPHYLTQTDYPAAAQALLEQVAKTASLELP--LTALSEAAEVIRAKIDEQVEA-SAEVAQVVAALERQYD---AFIDAQENRS-LLTRD--G
Rv2714            IQVPGSASNLLEYRMAQHGHEVVGFTVHVPHYLTQTDYPAAAQALLEQVAKTGSLQLP--LAVLAEAAAEVQAKIDEQVQA-SAEVAQVVAALERQYD---AFIDAQENRS-LLTRD--E
FRAAL1976         LQVPGSAGHLLEYELGRDGRDAMGFAVHVPHYLAQTTYPAATEVLLTSVSKATGLMLP--LDGLRSAAVAVQDEVNSQIAQ-GGEAAALVHALEEQYD---AYQRGRRGPSLPTIDAE-Q
NCgl1848          MTVPGSASLMLEKLLKDKGKNVSGYTVHVPHYVSASPYPAATLKLLQSIADSADLNLP--LLALERDAEKVHRQLMEQTEE-SSEIQRVVGALEQQYD--SELERYRNRHPQAVMPGE-S
CE1815            ISIPGSASLKLEKLLHTRGKNVAGYTVHVPHYVAASPYPAATLKLLQAVAETAELNLP--LLSIERDAEKVQRQLAEQTSD-SMEIQHVVGALERQYD--EELERYREKNPQAVLPGE-T
DIP1417           VTIPGSASLHIERLLNKNNIDVAGYTAHVPHYLSASAYPLATLKLLEAITTSTNLDFP--LKTLEADAQKTALQVEEQVAG-SQEIESVVKILEQQYD--EELEKYRERNPKALEIAE-D
cu0890            LVVPGAAALETEMQLSAAGFDAIGLTAHVPHYIAGSEYPEASYALLKAFADLADRELP--LKTLEKDMSRVQQQLAQQTQD-SHEIATVVGALEQQYD--KEVRRLQKKK-SSLLEPG-Q
Mmcs_2173         VQVPASVSSLLEFRMAQHGHEVVGYTVYVPHYLSQTAYPPAAESLLAEVAKTAALQIP--LTALGEAGAEVYTKINEQVEA-SVEVAQVVTALERQYD---AFVAAQENRS-LLAHD--E
RHA1_ro06805      LQVPGSASSLIELRMAQHGHESVGFSVHVPHYLAQTDYPGAAETLLENVSDVTDLDLP--LAALGEAAARVREQVDEHIAG-NEEVQTVVHALERQYD---AYVTAQEQQSTLLASE--E
nfa37830          LQVPGSASSLLEYRMAQHGHESLGFSVHVPHYLAQTAYPEAAQTLLEHVADNAGLELP--LAALGEAAARVREQVNEHIAG-NPEVETVVHALERQYD---SFVTAQERQSSLLAAD--G
Mflv_3951         VQVPGSASNLLEFRMAQHGYEVVGFTVHVPHYLAQTDYPSAAETLLAEVARTASLDIP--TAELTTAAAVVFDKINEQVTA-SAEVAQVVDALERQYD---AFVAAQENRS-LLARD--E
MUL_3357          IQVPASASNLLEYRMAQHGHEVVGFTVHVPHYLTQTDYPAAAQALLEQVAKTGSLDLP--LAALTDASAQVGAKIDEQVQA-SAEVAQVVAALERQYD---AFIDAQENRS-LLTRD--E
Franean1_5208     LSVPGSAGHLLEYEIGRSGADAMGFAAHVPHYLAQATYPAATEALLSSVSKSTGLLLP--LDGLRSAALEVRGEVDSQIAR-GGEAADVVKAIEEQYD---AFHRGREGEHLPVVDDS-E
MMAR_1998         IQVPASASNLLEYRMAQHGHEVVGFTVHVPHYLTQTDYPAAAQALLEQVAKTGSLDLP--LAALTDASAQVGAKIDEQVQA-SAEVAQVVAALERQYD---AFIDAQENRS-LLTRD--E
jk1100            MIVPGAAALETEKKLNRHGFETVGLTAHVPHYIAASDYPEAPLRLLQAFGKVANRELP--LKALEKELERVQHQLQDQVED-SQEIATVVSALEHQYD--AEMERMRRKRENNLLKPG-Q
Sare_1722         VQVPGSVGYLLEYRLGEQGRDALGFAAHVPHYVAQTEYPAAAEVLLSSVSRSTGLLLP--CDELRAATEAVRTEIDRQVAQ-TEDAAALVQALEEQYD---AFTRGRGQPNLLNTG-A-G
CMS_1627          TQVPANALHLLEHRLHDAGHPVAGFVLLVPHYLADTEFPLAAVAALESISAATGLIFP--TDRLREEGRDFVGRIDEQVAG-NQELARLVTTLEERYD---SYMEDTPLKSPLTD-ED-G
SSAG_03958        AQVPGSAESLVEFRLGQAGHDVLGVAAHVPHYVARSPYPDAALTALEAITAATGLVLPAVAHALRTEAHRTQTEIDRQIREGDEELVSLVQGLEHQYD---AAAGAETRGNMLAEP---A
Tfu_0926          IQVPGNIMSLLEYRLGQAGHDVIGYAVHVPSYLAQSEYPRAGLAVLEYIARVTGLVLP--TEKLAEEATRVDGEINRQVEA-SEEVQRVVRNLEAQYDNFMAVHNGLDVDALLAGD-E-S
SAV_6234          AQVPGSAESLVEYRLMEAGHDVLGVAAHVPHYIARSPYPDAALTVLEAITAATGLVLPGLAHSLRTEAHRTQTEIDRQIQEGDEELVALVQGLEHQYD---AAAGAETRGNMLAEP---V
Arth_1614         VEVPAAVGHILELRLSEAGRNIAGYVIHVPHYLAEAEYPNAAVAGLEYLGAATSLMLP--TDRLREAGREVGRQIAEQLEA-SEEVQQVVSRLETRYD---EKAEGTVRRSLLAD-EN-D
PPA1099           LQVPASFPVVLGQRLGETNHAVIGLASHVPHYLADLDYPESARALVEALRGATGLALP--INSLAVAANTVRAEIDTQVNN-SEELKAMLHALEEQYD---SRVAQRE-----LGTTQ-V
KRH_14390         FEVSASVSHLLELRLQENGIDHVGLSVHVPQYLAESYLPQAAVAALEYLSAVTGRTLP--TDELREASRDVIRQIDEQAGS-SPEVQAVIENMERRYD---DVVDASARRSILAGPDD-A
Krad_1509         AQVPGHASALLHLRGSQQGRDVLSVLAHVPHYLAGNDYPDAAAVLVRTLSGATGVGIP--VQSLQEAASRTRVEVDRQITE-SPEAASVVEALEEQYD---AIG-EEGRG-TGAAS---A
RSal33209_2429    VQIPSAIGHLLELRLSQSGRNIAGYAVHVPHYLADAEYPRAAVAALEYLGAAASLMLP--TDRLRESGRDVERQIADQVQT-SSEVQGVVRRLELQYD---ERQQDTPVRSLLAG-DG-D
ACTODO_01571      MQFPSPLSTFMQIRMGQRGIGGLVLLGAVPYYMADTGYPAASSALLTSFAKFADLSLP--VGDLEQGAAQDQENIAKLVEG-NPDISHTVSNLEEHFDAWTGGEGAIPLPGLGQRPSAGG
Rxyl_1077         YEGPTGITGVLHTLCAEAGLPAVSLWASMPHYLPAVPSAPGALALVESLAGLLGTEVE--TGDLERGAERYQEQVSAAVAR-DPDLSSYVKMLEERYD----------ASQEEGGGQIR
                     .                        :* *:.    . .    :  .       .      :         .:         :    :  :* :.*
```

The Gene ID of each sequence corresponds to the following genomes:

```
AAur_1758       ELPNAEDIGAAVEAYLARKDSAQ-----------------------------------
CMM_1644        ALPTADEIAAELEKFLARRRPGDGDAA-------------------------------
Acel_0028       NLPSADELAAELERFLAEQA-RRHRDDRDQ----------------------------
A20C1_03668     ELPSADEIAAELQKFLAIRRAGDDDSTTGLPL--------------------------
Lxx10170        ELPSADEIAAELENFLAFRRHGDEDNSRGDR---------------------------
MSMEG_2746      DLPSGDELGAEFERFLAQQAGEKFKDDKYKDGHGEGFGGRIRGTVTTTDDVPDQG-----
Mvan_2446       DLPSGEELGAEFERFLAQQAGEKKRKD-GDD-PREGL---------------------
Mb2733          DLPSGDELGAEFERFLAQQAEKKSDDDPT-----------------------------
SGR_5536        DLPSADEIGREFERFLAERE----GEG-------------------------------
ML1009          DLPSGDELGAEFERFLAQQAEKKRDDDLT-----------------------------
MAB_3033        ELPSGDELAGEFERFLAEQA--KFGDDPSGPSPEGPIL--------------------
SCO1997         EIPSADEIGREFERFLAERE----GDG-------------------------------
Strop_1737      ALPTADELGAELERFLAEQT-RPND-NPGG----------------------------
Francci3_1247   PLPTADELGDALERFLAEQT-EPDGPTPNP----------------------------
JNB_00545       DLPSADELGADIEEFLRNVSDD------------------------------------
Noca_2852       RLPTGEEIGKEFERFLAGLDRPGDDEPQG-----------------------------
MAP2831         DLPSGDELGAEFERFLAQQAEKKFDDDDQA----------------------------
Rv2714          DLPSGDELGAEFERFLAQQAEKKSDDDPT-----------------------------
FRAAL1976       KLPTADELGEALERFLAEQS-EPGGPNPLS----------------------------
NCgl1848        ELPSGDEIGAEFEKFLADLDDQGGSDHKETPEA-------------------------
CE1815          PVPSGDEIGAEFERFLANLDDSGDPDGPEDNGQ-------------------------
DIP1417         DLPDADELGEEFERFLANIDEENGSDD-------------------------------
cu0890          DIPTGEELGAEFEAFLARMNDAADTDSEADGEDPKKPGDSNDPHDDGEGDTQ--------
Mmcs_2173       DLPSGDELGAEFERFLAQQAGEKDKEDRYRDGFSDGE---------------DRS-----
RHA1_ro06805    DLPSGDELGAEFERFLAEQALQDGEGGDSDDEGGSPGGA-------------------
nfa37830        DLPSGEELGAEFERFLAEQGGYDG---DKD----------------------------
Mflv_3951       DLPSGEELGAEFERFLAQQAGEKKRKD-GDDQPREGL---------------------
MUL_3357        DLPSGDELGAEFERFLAQQAEKKFDDDDQP----------------------------
Franean1_5208   PLPTGEELGAALERFLAEQS-EPGGPQPT-----------------------------
MMAR_1998       DLPSGDELGAEFERFLAQQAEKKFDDDDQP----------------------------
jk1100          DIPTGEELGAEFEAFLQNMSDGDLSD--ENGADN--SADSDEQEEN------------
Sare_1722       SLPTADELGAELERFLAEQT-RPND-NPGG----------------------------
CMS_1627        ALPTADEIAAELEKFLARRRPGDGDAA-------------------------------
SSAG_03958      EIPSADEIGREFERFLAERE----GEG-------------------------------
Tfu_0926        RLPTADELGAELERFLAEHGRGSAEHDRGSEG--------------------------
SAV_6234        EIPSADEIGQEFERFLAERE----GEG-------------------------------
Arth_1614       ELPNADALGAAVEAYLAREEPRQ-----------------------------------
PPA1099         AVPDAEDIGAEVEDFLRSIDEDDGPSNDDPDTQGSGPRRSSDDTSDDDKPDYHPRRGGDL
KRH_14390       DLPDADDLGAAAEAFLAAQSDPDAHED-------------------------------
Krad_1509       FLPTADELGEEFERFLAGQEDEPDGDGPPGDRPGAG----------------------
RSal33209_2429  ELPDADELGAAIEAYLAERDDTAERDDTTG----------------------------
ACTODO_01571    EEKTPKDIGDVIEAYLAQVSRAQDEEIESVQRAPRTEESEEPSKPDTIEDVLARIEARRN
Rxyl_1077       HLPSGDELARELEDFLRRQRGEDEQ---------------------------------
                     . :.   : :*
```

AAur_1758: Arthrobacter aurescens TC1;
CMM_1644: Clavibacter michiganensis NCPPB 382;
Acel_0028: Acidothermus cellulolyticus 11B;
A20C1_03668: marine actinobacterium PHSC20C1;
Lxx10170: Leifsonia xyli subsp. xyli str. CTCB07;
MSMEG_2746: Mycobacterium smegmatis str. MC2 155;
Mvan_2446: Mycobacterium vanbaalenii PYR-1;
Mb2733: Mycobacterium bovis AF2122/97;
SGR_5536: Streptomyces griseus subsp. griseus NBRC 13350;
ML1009: Mycobacterium leprae TN;
MAB_3033: Mycobacterium abscessus;
SCO1997: Streptomyces coelicolor A3(2);
Strop_1737: Salinispora tropica CNB-440;
Francci3_1247: Frankia sp. CcI3;
JNB_00545: Janibacter sp. HTCC2649;
Noca_2852: Nocardioides sp. JS614;
MAP2831: Mycobacterium avium subsp. paratuberculosis K-10;
Rv2714: Mycobacterium tuberculosis H37Rv;
FRAAL1976: Frankia alni ACN14a;
NCgl1848: Corynebacterium glutamicum ATCC 13032;
CE1815: Corynebacterium efficiens YS-314;
DIP1417: Corynebacterium diphtheriae NCTC 13129;
cu0890: Corynebacterium urealyticum DSM 7109;
Mmcs_2173: Mycobacterium sp. MCS;
RHA1_ro06805: Rhodococcus sp. RHA1;
nfa37830: Nocardia farcinica IFM 10152;
Mflv_3951: Mycobacterium gilvum PYR-GCK;
MUL_3357: Mycobacterium ulcerans Agy99;
Franean1_5208: Frankia sp. EAN1pec;
MMAR_1998: Mycobacterium marinum M;
jk1100: Corynebacterium jeikeium K411;
Sare_1722: Salinispora arenicola CNS-205;
CMS_1627: Clavibacter michiganensis subsp. sepedonicus;
SSAG_03958: Streptomyces sp. Mg1;
Tfu_0926: Thermobifida fusca YX;
SAV_6234: Streptomyces avermitilis MA-4680;
Arth_1614: Arthrobacter sp. FB24;
PPA1099: Propionibacterium acnes KPA171202;
KRH_14390: Kocuria rhizophila DC2201;
Krad_1509: Kineococcus radiotolerans SRS30216;
RSal33209_2429: Renibacterium salmoninarum ATCC 33209;
ACTODO_01571: Actinomyces odontolyticus ATCC 17982;
Rxyl_1077: Rubrobacter xylanophilus DSM 9941.

Notes: Conserved residues are colored as such: purple, absolutely conserved residues; yellow, highly conserved. β strands and α helices are labeled in red and blue, corresponding to the ribbon diagram in Figure 2.
