# Supplementary material for: Structural and phylogenetic analysis of a conserved actinobacteria-specific protein (ASP1; SCO1997) from Streptomyces coelicolor
Source: BMC Struct Biol. 2009 Jun 10;9:40. doi: 10.1186/1472-6807-9-40 (PMC2714318; doi:10.1186/1472-6807-9-40)
Supplement: Additional file 4 — Alignment of all available ASP1 and ASP2 homologues from actinobacterial species. Conserved residues colored in Additional file 2 are also highlighted here. Unexpected amino acids at conserved positions are highlighted in cyan. [file 1472-6807-9-40-S4.pdf]

Additional file 4. Alignment of all available ASP1 and ASP2 homologues from actinobacterial species.

```
                                                                                                  β1               αA                β2
SCO1997          ------------------------------------MLDPQD---LYTWEPKGLAVVDMALA-QE--------------SAGL-VMLYHFDG-YIDAGETGDQIVDQVLDSLPHQVVARF
RSal33209_2429   ---------------MTDRCGRIR----MKEIKYVPLRDPDT---LYATNGA-------MLQDEG--------------LRGL-NLLMGFTG-FSDAGQVVSQINAELLDKLDAEPVAIF
MAB_3033         ------------------------MTSNEGVEPQPYKPDQSG---MYELEFP-----GPQLASAD--------------GRGP-VLVHALQG-FSDSGHAVKLAAAHLRQTLESELVASF
Noca_2852        ------------------------------------MPER-----LVHIVDE-----VPELDDAR--------------SRGALTMVLVLDG-FLDAGNAAGRAAQHLVDLSEGPVVATF
A20C1_03668      ------------------------------------MRDPAG---LYDIATD-------VAGVP----------------RGL-PLVAGLTG-FADAGGAVSQFSQYLLDTLERSVVASF
Krad_1509        ------------------------------------MLDPGS---LYSYEG------ERP----A--------------LVEP-PLVVALSG-FIDAGNAVRLSVEHLLATCRHEVVATF
CMS_1627         ----------------------------------------------------------------------------------------------------MSQVSEYLLSTLSHRDVLRF
Lxx10170         ------------------------------------MRDPGE---LFELTS--------ALEVP----------------DGL-PLVAGLTG-FADAGSGVSQLGTYLLATLHSEVVATF
nfa37830         ------------------------------------MDYESR---MYELEFP-----APQLSSAD--------------GSGP-VLVHGLEG-FTDAGHAVRLATTHLRESLESELVASF
PPA1099          ------------------------------------MDRQSN---RFSWHPG--------IVGPD--------------QEVS-ALITLVQS-FSDTGLVQARVRDAILSQLPHHELGEF
JNB_00545        ------------------------------------MQNPSD---LYRFEFD--------GPAPT--------------REPG-ALIVLLGA-FIDAGNIQRILGTHLVDTSEAEVVATF
DIP1417          ------------------------------------MNEREA--KMYELEFP-----APDVASDG---------------VDGPTLIIAMQG-YADAGLAVEASADHLLSALEHRPVAVF
Mflv_3951        ------------------------MADDAHDSGSRYQPEQTG---MYELEFP-----GPQLSTPD--------------GRGP-VLIHALEG-FSDAGHAIKLAAAHLKNSLDTELVASF
SGR_5536         ------------------------------------MPDPQS---LYEWEPKGLAVVDMALA-QE--------------SAGL-VMLYHFDG-YIDAGETGEQIVDGLLETLPHQVVARF
AAur_1758        MPSDVMSPGVMCWVPEPPRTLSLRKEHAVFERISGSLLDPES---LYARNIE-------TFHSPE--------------LRGL-NMVMGFTG-FADAGHVVRQINAELLDELGAEVVAMF
Mvan_2446        ------------------------MADDVHDPGHEYQPEQTG---MYELEFP-----GPQLSSPD--------------GRGP-VLIHALEG-FSDAGHAIRLSAQHLKDTLDTELVASF
MUL_3357         -----------------------MAHDQDPGEAQDYQPGQSG---MYELEFP-----APQLSTSD--------------GRGP-VLVHALEG-FSDAGHAIRLAATHLKDGLDTELVASF
Rv2714           -----------------------MARDQGADEAREYEPGQPG---MYELEFP-----APQLSSSD--------------GRGP-VLVHALEG-FSDAGHAIRLAAAHLKAALDTELVASF
RHA1_ro06805     ------------------------------------MDEQSK---MYELEFP-----APQLSSAD--------------GQGP-VLIHGLEG-FSDAGHAVKLATTHLRESLESELVASF
Sare_1722        ------------------------------------MLDPHE---LYELAD------DLP----D--------------LGQP-VLIQALSG-FVDAGNATRLAREQLLTSLDARPVARF
BLD_0447         ------------------------------------------MSEAAKDTGA--------------------------------VLIAAFEG-WNDACQAATNAVRHLVKRYESREIRHI
SSAG_03958       ------------------------------------MLDPQG---LYEWDAKGLAVADLALA-QD--------------SAGL-VMLYHFEG-YIDAGEAGEQIVERLLDTLPHQLVASF
BIFLAC_03242     -------------------------------------------------------------------------------------MVAAFDG-WNDACQSATDVIRHLVSRYRSLEIGHI
Tfu_0926         ------------------------------------MRDPAD---LYELRS------DLPEL-SE--------------P----VMLVSLDG-FVDAGAAGKQAVATLLDGLQHTELATF
SAV_6234         ------------------------------------MLDPQG---LYAWEPKGLAVVDMALA-QE--------------SAGL-VMLYHFDG-YIDAGETGDQIVDRLLDSLPHQVVARF
BL0722           ----------------------------------MNSSYSRGMSEAAKDTGA--------------------------------VLIAAFEG-WNDACQAATNAVRHLVKRYESREIRHI
Strop_1737       ------------------------------------MLDPHE---LYELTD------DLP----D--------------LGQP-VLIQALSG-FVDAGNATRLAREQLLTSLDARPVARF
CMM_1644         ------------------------------------MADADG---LYEIDTD-------IGEVP----------------TGL-PLVAGLTG-FSDAGSGVSQVSEYLLSTLSHRDVLRF
Francci3_1247    ------------------------------------MLDPDA---LYDLAE------DLAGGSID--------------LGRP-VMLEAMTG-VVDAGSAVSLAGEHLMTALDHRLLATF
MSMEG_2746       ------------------------MADNEN-PQQRYQPDQSG---MYELEFP-----APQLSSSD--------------GRGP-VLIHALEG-FSDAGHAIRLAAEHLKKSLDTELVASF
ML1009           ---------------------MSHSHYQDPDDEQHYQPGQPG---MYVLEFP-----APQLLASD--------------GRGP-VLIHALEG-FSDAGHAIRLAATHLKAALNTELVASF
Acel_0028        ------------------------------------MLDPTD---LYTISG------TPPARAPD--------------APL--ILVHALSG-YVDAGDAVRLTARHLLDTLDHREIARF
FRAAL1976        ------------------------------------MLDPQE---LYSLTE------DASDDPID--------------AHRP-IMLEALTG-VVDAGNAVSLAGEHLLTALDHRIVATF
Arth_1614        ----------------------------MLERISGSLLDPDA---LYVSNAE-------LFDNPE--------------LRGL-NLVMGFTG-FADAGHVVKQITAELLDTLESEVVAVF
Francean1_5208   ------------------------------------MLDPRE---LYEIRS------DLD----D--------------LGRP-VLIEAMTG-VVDAGGAVGLASEHLTTALQHERIVTF
KRH_14390        ------------------------------------MLDPTA---LYLGNPA-------LLGDPR--------------LRGL-PLVVALSG-YVEAGHLAAQIEAVVLDSMPSETVARF
BIFDEN_02062     -------------------------------------------------------------------------------------MLAAFEG-WNDACQAATNVIRHLVSRYDSQEIRHI
cu0890           ----------------------MPQ------DNSFGRRPRNA---MYEMEYP-----VPLEADSP---------------DEGIPMLIALQG-YADAGQAIAVSGNHLLEALESSVVASF
jk1100           ----------------------MHQHFENNGDEHNGRHERKAAGRMYEMEYP-----APIQFDRPSIADEATGLGSAVEGSEELPMLVALQG-YADAGQAVSSASSHLLAALEHSPVASF
NCgl1848         ------------------------------------MSDNND-RMYELEYP-----SPEVSGQT---------------AGGPTLIVALQG-YADAGHAVESSSSHLMDALDHRLIASF
cgR_1753         ------------------------------------MSDNND-RMYELEYP-----SPEVSGQT---------------AGGPTLIVALQG-YADAGHAVESSSSHLMDALDHRLIASF
MAP2831          -----------------------MTHRQDAGD--QYQPGQAG---MYELELP-----APQLSTSD--------------GRGP-VLVHALEG-FSDAGHAIRLAAKHLKAALDSELVASF
MMAR_1998        -----------------------MAHDQDPGEAQDYQPGQSG---MYELEFP-----APQLSTSD--------------GRGP-VLVHALEG-FSDAGHAIRLAATHLKDGLDTELVASF
ACTODO_01571     -----------------------------------MSINATD---LYADGPAAG--------------------------AKAKILLIHFDG-AIDAGAAGRMAIGQLLRSLHNERVATF
MAV_3608         -----------------------MTHRQDAGD--QYQPGQAG---MYELELP-----APQLSTSD--------------GRGP-VLVHALEG-FSDAGHAIRLAAKHLKAALDSELVASF
Mb2733           -----------------------MARDQGADEAREYEPGQPG---MYELEFP-----APQLSSSD--------------GRGP-VLVHALEG-FSDAGHAIRLAAAHLKAALDTELVASF
CE1815           ------------------------------------MSDNKE--SMYELEYP-----SPEVNGGG---------------SEGPTLIVALQG-YADAGHAVEASASHLMAALDHRLIASF
nfa31940         ----------------MNPSE-------------------TSDSELPTLRDP--------------------------------VLIAAFEG-WNDAGDAASGAVEHLELIWDAEPLAEL
RHA1_ro00860     ----------------MSSSEFPVTPAGRDDDTPDTEDAGATDSGVPVLRDP--------------------------------VLVAAFEG-WNDAGDAASGAVEHLELTWDAQPLAEL
Rv2125           ----------------MTPS--------------------EGNAPLPELHNT--------------------------------VVVAAFEG-WNDAGDAAGDAVAHLAASWQALPIVEI
MtubH3_05282     ----------------MTPS--------------------EGNAPLPELHNT--------------------------------VVVAAFEG-WNDAGDAAGDAVAHLAASWQALPIVEI
MT2184           ----------------MTPS--------------------EGNAPLPELHNT--------------------------------VVVAAFEG-WNDAGDAASDAVAHLAASWQALPIVEI
MUL_2357         ----------------MTPS--------------------DASRELPELHKT--------------------------------VVVAAFEG-WNDAGDAASDAVGHLAAIWNATPIVEI
Mvan_3475        ----------------MTPSDFSG----------------PKRSDLPDLRDT--------------------------------IVVAAFEG-WNDAGDAASDALEHLDAIWEAETIVEI
MAP1860          ----------------MTPPDGPPY------------GAPSGQAALPELHHT--------------------------------VVVAAFEG-WNDAGDAASDALEHLEAIWEADPILEI
MAB_2128c        ----------------MTLSD-------------------MAQNSLPVLRDP--------------------------------IVVAAFEG-WNDAGDAASAALEHLDTTWQATPLMTI
Arth_2169        ----------------MNSFDGDTSEPGA-----TPEPERLLQPVPEGQRIT--------------------------------VMLAAFEG-WNDAGEAASDALRYLNKLWGGKKVASI
ML1306           --------------------------------------------------------------------------------------MAAFEG-WNDASDAASGALEHLNAVWEADPIVEI
Mflv_3052        ----------------MTPSDFGG----------------PKRSDLPDLRDT--------------------------------IIVAAFEG-WNDAGDAASDALEHLDAIWEAETIVEI
SGR_5841         ---------------------------------------MIELESVPELIDP--------------------------------VMVAAFEG-WNDAGDAASTAVGHLDREWKGEVFAAL
RSal33209_2043   ----------------MNRFDDAASE------------GKLLPSAVEGQRIT--------------------------------VMVAAFEG-WNDAGDAASDALKYLNKLWGAVKVGRI
SCO1662          ---------------------------------------MIELEGVPELVDP--------------------------------VMVAAFEG-WNDAGDAASTAVAHLDREWKGEVFAAL
Lxx08270         -----------------------------------------MTDANSILGGR--------------------------------ILVVAFEG-WNDAGEAASGAVKTLKDQLDVVPVAEV
A20C1_02494      -----------------------------------------MTNT-PFANGR--------------------------------LLVVAFEG-WNDAGEAASGAVRSLKEQLDVYPIAEV
BlinB01000025    -------------------------------------------------------------------------------------MFIAFEGQDADASEAASSLVKDLIDAWELNDSEIL
SSAG_04705       ---------------------------------------MIELEGVPELIDP--------------------------------VMVAAFEG-WNDAGDAASGAVAHLDREWKGEVFAAL
Rxyl_1077        -----------------------------------MDERYLDLEWRPELERP--------------------------------VLVSAFTG-WNDAAEAASMALSALGNAWGVRRFGRF
Noca_2630        ---------------------------------------MIELEDVPDLVDP--------------------------------VVIAAFEG-WNDAADAASSVIDHLIRVWGARTVGAV
TWT317           ---------------------------------MGTIGVCNRGGVASFLDCR--------------------------------VLIAAFED-WGDAGKVSTTALRMLMEIFHAKPVKEI
Mjls_3162        ----------------MTPSDFGP----------------EKGPDLPPLRDA--------------------------------VVVAAFEG-WNDAGDAASDALEHLDAIWEAETIAEI
MSMEG_4186       ----------------MTSSYPPV----------------GRPADLPELENA--------------------------------IVVAAFEG-WNDAGDAASDALDHLDAIWEAETIVEI
Sare_2324        ---------------------------------------MTEFDGLPVLRSP--------------------------------VAIAAFEG-WNDAADASTAAVEHLEQVWQARPVTEL
FRAAL2860        ---------------------------------------MIEFSGVGQLRSP--------------------------------VVVAAFEG-WNDAADASSAAVEHLETAFKARVIGAI
SAV_6650         ---------------------------------------MIELEGVPELIDP--------------------------------VMVAAFEG-WNDAGDAASTAVAHLDREWKGEVFAAL
Strop_2170       ---------------------------------------MTEFDGLPVLRSP--------------------------------VAIAAFEG-WNDAADASTAAVEHLEQVWQARPVTEL
Francci3_2640    ---------------------------------------MIEFSGVGQLRSP--------------------------------VVVAAFEG-WNDAADASSAAVEHLETAFKARVIGAI
Acel_1173        ---------------------------------------MIEFSELRPLRNP--------------------------------VMITAFEG-WNDAAEAATDAIETLELAWHAEPIAEI
Tfu_1826         ---------------------------------------MPELDSVSELVEP--------------------------------VMVAAFEG-WNDAGEAASAVIDHLTDVWGATELVSL
Krad_1842        ----------------MPEQPVDPR--------------AESAPAPVVLRDP--------------------------------VVIAAFEG-WNDAGEAASQALAHLHEVWGAVDLVEF
KRH_13910        ----------------MSMLDSNSQHPG-------TGPFGVVRDGLDQEPVT--------------------------------VLIAAFEG-WNDAGDAATDTLKLIREHLGAREVFEV
Francean1_4890   ---------------------------------------MIEFSGVGQLRSP--------------------------------VVVAAFEG-WNDAADASSAAVEHLENVFGARVIGAI
SACE_2233        ----------------MTDRDPTP---------------AEDSREQPELTDP--------------------------------IVVAAFEG-WNDAGDAASSAIEHLQLTWDATPLSEI
JNB_03080        ---------------------------------------MIELDDLPELENA--------------------------------VLIAAFEG-WNDAGEAASGVIEHLAQLWDAEPVAAL
CMM_1693         -----------------------------------------MADAERFVSGR--------------------------------LLVVAFEG-WNDAGEAASGAVRALKDLLDLEAVSSV
CMS_1670         -----------------------------------------MADAERFVSGR--------------------------------LLVVAFEG-WNDAGEAASGAVRALKELLDLEAVSSV
PPA1200          ---------------------------------------------------------------------------------------MAFGG-WNDAGTAATQAASLIAEYSDAKIWRTI
AAur_2170        ----------------MNSVDGTPEGQDM-----TAEPERFLKEPADGQRVT--------------------------------VMLAAFEG-WNDAGEAASDALHYLNKLWDGKKVATV
TW455            --------------------------------------------MASFLDCR--------------------------------VLIAAFED-WGDAGKVSTTALRMLMEIFHAKPVKEI
BIFADO_01161     ------------------------------------------MCEESAMPKT--------------------------------ILIAAFEG-WNDASQAATNVVRHLVSRYESQEVRHI
BAD_0824         ------------------------------------------MCEESAMPKT--------------------------------ILIAAFEG-WNDASQAATNVVRHLVSRYESQEVRHI
```

```
                                      β3        β4           β5                          β6          αB            β7            β8  αC
SCO1997          DHDRLVDYRARRPLLTFK--RDTWSDYEEPTIE----------------VRLVQDATGAPFLFLSGPEPDVEWERFAAAVGQIVERLGVRLSVSFHGIPMGVPHTRPVGITPHGSRTDL
RSal33209_2429   DIDQLIDYRSRRPQISFV--EDHLTDYRQPEMV----------------LYRMVDGLGQPFLFLAGNEPDLQWERFSAAVVALVERLDVNLVAWVHSIPMPVPHTRPIGVTVHGNRPDL
MAB_3033         AIDDLLDYRSRRPVMTFK--SDHFTEYATPELN----------------LYALKDTKGTPFLLLAGLEPDLKWERFVNAIRLLAEQLGVRKTIGLGAIPMAVPHTRPITLTAHGNDRKT
Noca_2852        DVDEFHDYRARRPPMSFV--RDHYDAYDAPRLV----------------VRLLADTGGTPYLLLHGPEPDNRWEAFCRAVREVVERFGVSRVVGMGSVPMAVPHTRPIAITHHANSPEL
A20C1_03668      DNDALLDYRARRPTIYFD--QDHLSDYRPAKLE----------------VYLAKDELGQPFLLLTGYEPDFQWERFSAAVLRLVEKFDVKSTTWVHAIPMPVPHTRSIGVTVSGNRSEL
Krad_1509        DVDQLHDYRARRPTMTFG--GQDWQDYEPPRLQ----------------VHRVHDEDGSSLLLLTGPEPDVQWERFTAAVWQLADELGVGATIVLSAAPTAVPHTRPSGVTASASRPEL
CMS_1627         DTDTLLDYRARRPTIYFD--QDHLADYRPARLA----------------LYLAHDEIGQPFLLLTGFEPDFRWEAFTAAVLGIVDRYRVSTTTWVHAIPMPVPHTRDINVTVSGNRTEL
Lxx10170         DADILLDYRARRPIIYFD--QDHLDGYQPSTLK----------------LYLVYDELRQPFLLLSGFEPDFRWEAFTEAVLGLMERFRVKSVTWVHAIPMPVPHTRPIGVTVSGNRAEL
nfa37830         DVDELLDYRSRRPLMTFK--TDHFSDYAEPELN----------------LWALRDTAGTPFLLLAGLEPDLRWEKFTTAVRLLAEQLGVRRSIGLSAIPMAIPHTRPLGITAHSSDRSL
PPA1099          DIDLLLDHRDSRQPIIFD--TDHFEGYERPRLV----------------LHEVTDQLGQAFLVLEGPEPALGWESLVSSLTSLVDSMGIRLTVITDSIPIPTPHTRPAIVTRWASRPEL
JNB_00545        DVDALLDYRGRRPAMVFD--TNRWESYADPSMV----------------LYRMSDRDGQTYYLLTGPEPDFQWERTIEAIRQLIQLLGISLVVTSHGIPMGVPHTRPVGMTAHATDPRL
DIP1417          NNDELIDYRSRRPAVTID--HNIVVDANELKLS----------------MDVLRDSNGTPFLLLSGPEPDLRWDAFSQAVADLVRRFGVRRTICIYAAPMTVPHTRPLIVSAHGNSLDL
Mflv_3951        AIDELLDYRSRRPLMTFK--TDHFTHYDEPELN----------------LYALHDSVGTPFLLLSGMEPDLRWERFVTAIRLLAERLGVRRVIGLGSIPMAVPHTRPMTLTAHSNDKEL
SGR_5536         DHDRLVDYRARRPLLTFR--RDRWTSFEAPALE----------------VRVVQDATGAPFLLLSGPEPDVEWERFAAAVEQIVERLGVRLAVNFHGIPMGVPHTRPVGITPHGNRTDL
AAur_1758        DADQLIDYRSRRPHISFV--EDHLQDYQAPKLG----------------LYKLNDGLGQPFLLLAGFEPDLQWERFSRAVVGIVEELDVNLVTWIHSIPMPVPHTRPVGVTVHGNMPDL
Mvan_2446        AIDELLDYRSRRPLMTFK--TDHFTHYDQPELN----------------LYALRDTAGTPFLLLAGLEPDLRWERFITAVRLLSERLGVRRVIGLGSIPMAVPHTRPMTLTAHSNDKEL
MUL_3357         AIDELLDYRSRRSMMTFK--TDHFTKYDDPELS----------------LYALRDSVGTPFLLLAGMEPDLKWERFITAVRLLAERLGVRQTIGLGTVPMAVPHTRPVTMTAHSNNKEL
Rv2714           AIDELLDYRSRRPLMTFK--TDHFTHSDDPELS----------------LYALRDSIGTPFLLLAGLEPDLKWERFITAVRLLAERLGVRQTIGLGTVPMAVPHTRPITMTAHSNNREL
RHA1_ro06805     AVDELVDYRSRRPTMTFK--ADHFSDYDQPELN----------------LYALKDTAGTPFLLLAGMEPDLRWERFTTAVRLLAEQLGVRRTVGINAIPMAIPHTRPLGVTAHSTNKDL
Sare_1722        DLDQLFDYRSRRPVMTFV--EDHWESYDAPALE----------------LHLLRDDADTPFLLLTGPEPDLQWERFVAAVAGLATRLDVRLTVGLNAIPMAVPHTRRTGVTAHATRREL
BLD_0447         RCDDFYDYQVARPMLCHVS-GRTNLIWPQTTFY-----------------DITLDAGKHIYAQIAPEPNYRWKEYCSQSLAIADELDINRIITLGSMFSDCPHTRPLPIAVSDGDCQC
SSAG_03958       DADRLVDYRARRPLLTFQ--RDHWSEFEEPRLE----------------VRLVQDATGAPFLLLSGPEPDVEWERFAVAVRQIVERLGVRLSVNFHGIPMGVPHTRPVGITPHGNRTDL
BIFLAC_03242     NKDGYYDYQSARPMQCTIQ-GSRCILWPQTVFY-----------------DIAVSDSLHILAQLGPEPNYQWLEYCRGSLRFAEQYDVECIVTLGSMLAECPHTRPLPIDISNNGKPL
Tfu_0926         DIDGLLDYRSRRPVMIFN--ETTWVSYAEPKLS----------------LTLLHDLEGTPFLLLHGLEPDRRWEGFTAAVRQLVERLSVRLTVCFYGIPMAVPHTRPVTATPHATRPEL
SAV_6234         DHDRLVDYRARRPLLTFK--RDRWTEYEEPTLD----------------VRLVQDTTGAPFLLLSGPEPDVEWERFAAAVQQIVERLGVRLSVNFHGIPMGVPHTRPVGLTPHGNRNDL
BL0722           RCDDFYDYQVARPMLCHVS-GRTNLIWPQTTFY-----------------DITLDAGKRIYAQIAPEPNYRWKEYCSQSLAIADELDVNRIITLGSMFSDCPHTRPLPIAVSDGDCQC
Strop_1737       DLDQLFDYRSRRPVMTFV--EDHWESYDAPALE----------------LHLLRDDADTPFLLLTGPEPDLQWERFVAAVAGLSARLDVRLTVGLNAIPMAVPHTRRTGVTAHATRREL
CMM_1644         DTDTLLDYRARRPTIYFD--QDHLADYRPARLA----------------LYLAHDEIGQPFLLLTGFEPDFRWEAFTAAVLGIVDRYRVSTTTWVHAIPMPVPHTRDINVTVSGNRTEL
Francci3_1247    DIDQLLDYRSRRPTMVFS--EDRWESYEDPVLA----------------LYLLRDEAGTPFLLLAGPEPDLQWKRFTVALRGLVARLGVRLTVGLNAIPMAVPHTRPLVVSAHATRKDL
MSMEG_2746       AIDELLDYRSRRPLMTFK--TDHFTAYEEPELN----------------LYALHDTVGTPFLLLAGLEPDLRWERFITAVRLLAEQLGVRQVIGLGTIPMAVPHTRPVNLTAHSNNKEL
ML1009           AIDELLDYRSRRPLMTFK--TDHFTHYDDPELS----------------LYALRDSVGTPFLLLAGMEPDLKWERFITAVRLLAERLGVRQTISLGTVPMAVPHTRPITLTAHSNNGEL
Acel_0028        DIDQLFDYRARRPPMLFD--EDHWAAYDAPELV----------------VHAVTDGGGREFLLLTGPEPDTQWERFVAAVCTLLERYGVDVTVGLNAIPLATPHTRPIGVTRHATSQDL
FRAAL1976        DVDQLLDYRSRRPTMIFS--EDHWESYTDPVLA----------------LYQLRDESDTPFLLLTGPEPDLQWKRFTAAVRGLVARLGVRLTVGLNAVPMAVPHTRPATITAHGSSKEL
Arth_1614        DADQLIDYRSRRPHVTFV--EDHLQDYQAPTLA----------------LYRLVDGLGKPFLFLAGFEPDLQWERFARAVVRIVEHLDVNLVTWIHSIPMPVPHTRPVGVTVHGNRPEL
Franean1_5208    DVDQLMDYRSRRPPMVFY--EDHWESYDDPVLA----------------IELLHDEAGTPFLLLCGPEPDLHWKRFTKAVQAVMAELGVRMSVGLNAIPMAVPHTRPCGVTAHATRKEL
KRH_14390        DIDQLYDYRSRRPRVTFA--QDHFEDWEAPELS----------------LHVVTDGLDRHFLMLTGPEPDTQWERFASAVVGLAQRLQVSLVAVVGGIPMPVPHTRPVLVTAHGPRADL
BIFDEN_02062     NDEGFYDYQVARPMICSVQ-GRRRIIWPQTTFY-----------------DITISPETHFLAQIAPEPNYRWEEYCRKTLRIAEDYDVNEIVTLGSMFADCPHTRPLPLDISKGDCEC
cu0890           NLDDLLDYRARRPSITID--ENRITKSDKLSLD----------------VRIVKDVNDRPFLLVAGPEPDLKWEAFGHAIVELAQRANVSRAISFYSAPMTVPHTRPLVVSAHASEPSL
jk1100           KVDELIDYRSRRPGVTID--NSKVMDSDDVRLD----------------LHLLKDTQGRPFLLLSGLEPDLKWGAFSDAVVDLARRGQVGMVASLYSAPMTVPHTRPLVISAHSSDSSL
NCgl1848         NNDELIDYRSRRPVVVIE--HNEVTSMDELNLG----------------LHVVRDNDNKPFLMLSGPEPDLRWGDFSNAVVDLVEKFGVENTICLYAAPMTVPHTRPTVVTAHGNSTDR
cgR_1753         NNDELIDYRSRRPVVVIE--HNEVTSMDELNLG----------------LHVVRDNDNKPFLMLSGPEPDLRWGDFSNAVVDLVEKFGVENTICLYAAPMTVPHTRPTVVTAHGNSTDR
MAP2831          AIDELLDYRSRRPLMTFK--TDHFTHYDDPELS----------------LYALRDSVGTPFLLLAGLEPDLKWERFITAVRLLAERLGVRQTIGLGTVPMAVPHTRPITMTAHSNNPEL
MMAR_1998        AIDELLDYRSRRPMMTFK--TDHFTKYDDPELS----------------LYALRDSVGTPFLLLAGMEPDLKWERFITAVRLLAERLGVRQTIGLGTVPMAVPHTRPVTMTAHSNNPEL
ACTODO_01571     DADALMDYRSHRPIVTVDN-WASSPDMVMPETV----------------LDLVEDDMGNPILVLHGAEPDAHWESFTNAINELCERAGVEITFSLHGVPSGVPHTRPTPVHVQATDASL
MAV_3608         AIDELLDYRSRRPLMTFK--TDHFTHYDDPELS----------------LYALRDSVGTPFLLLAGLEPDLKWERFITAVRLLAERLGVRQTIGLGTVPMAVPHTRPITMTAHSNNPEL
Mb2733           AIDELLDYRSRRPLMTFK--TDHFTHSDDPELS----------------LYALRDSIGTPFLLLAGLEPDLKWERFITAVRLLAERLGVRQTIGLGTVPMAVPHTRPITMTAHSNNREL
CE1815           NNDELIDYRSRRPTVVID--NNEVTEMDELQLG----------------LHVVRDSDNKPFLVLSGPEPDLRWGAFTEAVSDLVDKFGVDQTICLYAAPMTVPHTRPTVVSAHGNCAEL
nfa31940         DSEDYYDYQVNRPTVRQVDGVTREIQWPSTMLS----------------VCSPP-GTDRDVVLLRGIEPNMRWRSFCADLLEFVEQLGVQTVVILGALLADTPHTRPVPVTGSAYSKEA
RHA1_ro00860     DSEEYYDYQVNRPTVRQVDGVTREIVWPSTMLS----------------VCSPP-GSDRDVVLLRGIEPNMRWRSFCEDLLEIDLQLQINTVVILGALLADTPHTRPVPVTGSAYSSEA
Rv2125           DDEAYYDYQVNRPVIRQVDGVTRELQWPAMRIS----------------HCRPP-GSDRDVVLMCGVEPNMRWRTFCDELLAVIDKLNVDTVVILGALLADTPHTRPVPVSGAAYSAAS
MtubH3_05282     DDEAYYDYQVNRPVIRQVDGVTRELQWPAMRIS----------------HCRPP-GSDRDVVLMCGVEPNMRWRTFCDELLAVIDKLNVDTVVILGALLADTPHTRPVPVSGAAYSAAS
MT2184           DDEAYYDYQVNRPVIRQVDGVTRELQWPAMRIS----------------HCRPP-GSDRDVVLMCGVEPNMRWRTFCDELLAVIDKLNVDTVVILGALLADTPHTRPVPVSGAAYSAAS
MUL_2357         DDEAYYDYQVNRPVIRQIDGVTRELEWPAMRIS----------------HCRPP-DSDRDVVLMCGLEPNMRWRTFCAELLAIIEKLNVDTVVILGALLADTPHTRPVPVSGAAYSAES
Mvan_3475        DDEAYYDYQVNRPVIRQVDGVTRELVWPSMRIS----------------HCRPP-GSDRDIVLMHGVEPNMRWRTFCAELLAIAEKLNVQTVVILGALLADTPHTRPVPVSGAAYSPDS
MAP1860          DDEAYYDYQVNRPVIRQVDGVTRELVWPAMRIS----------------HCRPP-GSDRDVVLMHGVEPNMRWRTFCAELVAIADKLNVDTVVILGALLADTPHTRPVPVSGAAYSPES
MAB_2128c        DDEDYYDYQVNRPVVRLVDGVTRELEWPGMHIS----------------YCSPP-GSQRDVVLMHGAEPNMRWRTFCNRLLAIADRLDVSTVVILGALLADTPHTRPVPVSGTAYSPEA
Arth_2169        DADEYYDFQFTRPTIRRTSSGERKIKWPSTRIY----------------KASAP-DANVDVIFVQGTEPSYKWRAYTAELLVHAEALHVDYVILVGALLADVPHSRPIPVSTSTDDSAL
ML1306           DDEAYYDYQVNRPVIRQVDGVTRELVWPAMRIS----------------YCRPP-GSDRNVVLMHGVEPNMRWRTFCTELLTIADRLNVDTVVILGALLADTPHTRPVPVSGAAYSPES
Mflv_3052        DDEAYYDYQVNRPVIRQVDGVTRELVWPSMRIS----------------HCRPP-GADRDIVLMHGVEPNMRWRTFCAELLNIADKLNVQTVVILGALLADTPHTRPVPVSGAAYSADS
SGR_5841         DAEDYYDFQVNRPTVWLDG-GTRKITWPTTRLS----------------VVRVGGDKPRDLVLVRGIEPSMRWRSFCNEILGFAHELGVEMVVILGALLGDTPHTRPVPVSGVTSDADL
RSal33209_2043   EADEYYDFQFTRPVIKRTASGARKVKWPTTKLS----------------KVSLP-GSAVDVVFVYGIEPSYKWCAYTDELLEHAEDLNVDCLILVGALLADVPHSRPIPVTSSSDDDRF
SCO1662          DAEDYYDFQVNRPTVFMED-GVRKITWPTTRLS----------------VVRVGGDKPRDLVLVRGIEPSMRWRSFCNELLGFAHELGVELVVVLGALLGDTPHTRPVPISGTTSDADL
Lxx08270         DPELYFDFQFNRPVVA-DDDGRRLIWPSAEILGPARPGD------TGDARLDATGANAGNIFLLLGTEPSRSWRSFTAEIMDAALASDIGAIVFLGAMLADVPHTRPISIFASSENAAV
A20C1_02494      DPEEYFDYQFNRPVVSYDDDGNRALTWPSVAIYGPSRPNAERPESIAADAELQLSANNQSNIYLLLGTEPSRSWKAFTRDILETVVENGITSIVFLGAMLADVPHTRPISVFTSSENPEV
BlinB01000025    DTDGFYEFRFSEPEIVRDESNRATIAWPGIEVH------------------RGQHMGTPITIIQGHEPGLKWREFLSLILAEVTAED--CVVLLGGLHAEVPHTRPLPVVANTEDIRL
SSAG_04705       DAEDYYDFQVNRPTVWLDN-GVRKITWPTTRLS----------------VVRIGGAKPRDLVLVRGIEPSMRWRSFCNEILGFAHELGVEMVVILGALLGDTPHTRPVPVSGVTSDADL
Rxyl_1077        DPEEFFDYQSTRPQVKLVDGVTRKIEWPENALYAT-------------AAGGVEALGGRGAVLLSGPEPNLRWRTFSETVVGLAREVGMEMVVTLGALLADVPHSRPVPVAANAQEASL
Noca_2630        DPEDFYDFQVNRPVVGTDELGHRRLTWPSTQIA----------------IASPP-DLDRDVILIRGIEPNMRWRQFCADELLAACDELGGELVVTLGALLADTPHTRPIPVSGTATEPEL
TWT317           DADDYLDYHLYRPRCESKD-GVRKIYWPAPTVYKMS------------NEPGVPSGSAEDIYFLISPEPNMRWRKFAVEIVDYAKANNISGIIFLGSMLTDIPHTRPFPVFLTSEEPKV
Mjls_3162        DDESYYDYQVNRPVIRQVDGVTRELVWPSMRIS----------------HCRPP-GADRDIVLMHGVEPNMRWRTFCAELLNIADKLNVQTVVILGALLADTPHTRPVPVSGAAYSPES
MSMEG_4186       DDESYYDYQVNRPVIRQVDGVTRELVWPSMRIS----------------YCRPP-GSDRDVVLMHGVEPNMRWRTFCAELLAIAEKLNVQTVVILGALLADTPHTRPVPVSGAAYSAES
Sare_2324        DPEDFYDFQVSRPTITMSDGETRRVEWPTTRFM----------------AASPA-GTERDVVLIRGIEPSMRWRTFCEQVLEICHSLEVERVVLLGALLADVPYTRPLPISGSASDKGA
FRAAL2860        DPDDYYDFQVNRPTVSGTDGASRKITWPTTRLS----------------VARPP-KSETDLVLIRGLEPNMRWRGFTDELIEAVHALGSDMVISLGALLADSPHTRPVPISGTAGDAAT
SAV_6650         DAEDYYDFQVNRPTVWLDG-GVRKITWPTTRLS----------------VVRVGGEKPRDLVLVRGIEPSMRWRSFCNELLAFAHELGVELVVVLGALLGDTPHTRPVPVSGVTSPDL
Strop_2170       DPEDFYDFQVSRPTIAMSDGETRRVEWPTTRFT----------------VASPA-GTERDVVLIRGIEPSMRWRTFCEQVLEICHSLEVERVVLLGALLADVPYTRPLPISGSASDKGA
Francci3_2640    DPDDYYDFQVNRPTVSGTDGASRKISWPTTRLS----------------VARPP-KSETDLVLIRGLEPNMRWRGFTDELIEAVHALGSDMVITLGALLADSPHTRPVPISGTAGDAAT
Acel_1173        DPEEYYDFQVNRPTVALIDGVSRRIAWPTSRLS----------------IASPP-ESGRDVVLLRGIEPNMRWRTFCAELLDAAGRLGVTEVVNLGALLSDSPHSRPVPVTGSATDSAA
Tfu_1826         DSEEYYDFQVTRPQVTAVNGAHQGIHWPGVKVL----------------LTRSP--EGRDVVLVRGDEPSMRWRGFASDLLSIARELGVRRVVLLGALLADVPHTRPVPVTGMFSHPEL
Krad_1842        DPELYHDFQVNRPTVHYDDDGRRRLTWPTTRVS----------------YALLP-DAPRDVLLVRGIEPSMRWRTYAREVVEQAQAHGASLVVTLGALLADVPHTRPLPITSSSEDPDL
KRH_13910        ASDDYYDYQYSRPVIRRSLSGEQKLQWPSTRLS----------------KASVP-GTNLDVVLLHGWEPSYRWRTFTAELLEQATALDVDFVIMLGSLLADVPHTRPIPVTLTSDTEEL
Franean1_4890    DPDDYYDFQVNRPTVVSGADGASRKIAWPTTRLS---------------VARPA-KSESDLVLVRGLEPNMRWRGFTDELIEAVHALGSDMVITLGALLADSPHTRPVPISGTAGDPAT
SACE_2233        DPDPYYDFQVSRPTVKLVDGITRKITWPTTRLS----------------VCRPP-GSGRDIVLVHGIEPNMRWRAFCSELVDQVERIGASTVVSLGALLADSPHTRPVPVTGAAYDSAS
JNB_03080        DPEDYYDFQVNRPRV-VSEGGQRRISWRTTRIL----------------VATNT-SLGRDVILVQGVEPSFRWRSFAIEIMELAQAADVTTVITLGALLADVAHTRPIQVTVTSDSEDT
CMM_1693         DPEDYFDFQFNRPTIGFAEDGTRELEWPGATLYGPKDPRRP-AQDLAADAQLGVSGDNGGSIHLLLGVEPSRHWTAFTAEVLDAARAAGVEGVVLLGALLADVPHTRPISVYSTSENASV
CMS_1670         DPEDYFDFQFNRPTIGFAEDGTRELEWPGATLYGPKDPRRP-AQDLAADAQLGVSGDNGGSIHLLLGVEPSRHWTAFTTEVLDAARAAGVEGVVLLGALLADVPHTRPISVYSTSENAAV
PPA1200          DGEVFHDFRQTRPAITVMN-DLEIVSWPSTAIR------------------VGRWQGQPIVVVDGPEPSLRWRTYCFRLLRRLKLNPAGLVVLGAMATDVAHTRQLPVMVSATDPNL
AAur_2170        DAEEYYDFQFTRPTVRRTSSGARKVKWPSTRIY----------------KAEVP-DSNVDVVLVLGTEPSYRWRAYTTELLVHAEALKVDSVILVGALLADVPHSRPIPVSTTTEDSSL
TW455            DADDYLDYHLYRPRCESKD-GVRKIYWPAPTVYKMS------------NEPGVPSGSAEDIYFLISPEPNMRWRKFAVEIVDYAKANNISGIIFLGSMLTDIPHTRPFPVFLTSEEPKV
BIFADO_01161     DNEGFYDYQVARPMICSVQ-GRKRIIWPQTTFY-----------------DIAVSPMLHLLAQIAPEPNYRWEEYCRQTLRVAEDYDVSDVITLGSMFDECPHTRPLPLDISKSGCEC
BAD_0824         DNEGFYDYQVARPMICSVQ-GRKRIIWPQTTFY-----------------DIAVSPMLHLLAQIAPEPNYRWEEYCRQTLRVAEDYDVSDVITLGSMFDECPHTRPLPLDISKSGCEC
                   :   :.:  .                                                     . **   *                                   .:.:*
```

```
                         αD              β9              αE                 αF                 αG
SCO1997          VP-GHRSPFEEAQVPGSAEALVEYRLAQAGHDVLGVAAHVPHYVARSA-YPDAALTVLEAITAATGLVLPGIAHSLRTDAHRTQTEIDRQIQEGDEELIALVQGLEHQYD----AAAGAE
RSal33209_2429   IE-GMSSWKATVQIPSAIGHLLELRLSQSGRNIAGYAVHVPHYLADAE-YPRAAVAALEYLGAAASLMLP--TDRLRESGRDVERQIADQVQTSSE-VQGVVRRLELQYD--ERQQDTPV
MAB_3033         LD-EHPGWIDEVQVPGSASNLLEFRLAQHGHDVVGFVAHVPHYLAQTD-YPEASQRLLEEVARTGDLDLP--LQELSEAAAKVRNQINEQVEGSE-VAQVVQALERQYD---AFVAAQE
Noca_2852        IT-GESPWRGELRIPSSAQALLEVRLGEWGHDAMGFVAHIPHYLAQMD-YPRASAALLEQVEIAGRLTVD--LSGLRAEAEDREAEIARYLAANEE-VAEVVAALERQYD---AFERAEE
A20C1_03668      ID-SLSVWKPHTQVPANAMHLIEYRLQELGHPTAGFVLLIPHYLADTE-WPTAAVTALESVSAATGLIFP--TDRLREDGREFVAKIDSQVESNQE-LARLVGTLEERHD--NYMEENPM
Krad_1509        LD-GYRTWDVEAQVPGHASALLHLRGSQQGRDVLSVLAHVPHYLAGND-YPDAAAVLVRTLSGATGVGIP--VQSLQEAASRTRVEVDRQITESPE-AASVVEALEEQYD--AIGEEGRG
CMS_1627         ID-ALSVWKPHTQVPANALHLLEHRLHDAGHPVAGFVLLVPHYLADTE-FPLAAVAALESISAATGLIFP--TDRLREEGRDFVGRIDEQVAGNQE-LARLVTTLEERYD--SYMEDTPL
Lxx10170         IE-AMSIWKPQTQVPANALHLLEFRLQPLSYPIAGFVLLIPHYLADAE-YPAAAITGLESISAATGLIFP--TDRLREDDRQFVANINEQVAGNAE-LGRLVGTLENRHD--SYMEDNQP
nfa37830         IA-DHQRWPGELQVPGSASSLLEYRMAQHGHESLGFSVHVPHYLAQTA-YPEAAQTLLEHVADNAGLELP--LAALGEAAARVREQVNEHIAGNPE-VETVVHALERQYD---SFVTAQE
PPA1099          IL-GSTSPFGRLQVPASFPVVLGQRLGETNHAVIGLASHVPHYLADLD-YPESARALVEALRGATGLALP--INSLAVAANTVRAEIDTQVNNSEE-LKAMLHALEEQYD---SRVAQRE
JNB_00545        VG-GKESPFGQIQVPASFPALLELRLGEAGRDALGFAIHVPQYLAQAE-WAEGALAALNAITDATGLNLP--NDDLIAKAGINNREIASELNDNAD-AGQIVSALEQQYD---AFVEGQE
DIP1417          IK-HHFSIDSKVTIPGSASLHIERLLNKNNIDVAGYTAHVPHYLSASA-YPLATLKLLEAITTSTNLDFP--LKTLEADAQKTALQVEEQVAGSQE-IESVVKILEQQYD-EELEKYRER
Mflv_3951        IA-EHQPWVGEVQVPGSASNLLEFRMAQHGYEVVGFTVHVPHYLAQTD-YPSAAETLLAEVARTASLDIP--TAELTTAAAVVFDKINEQVTASAE-VAQVVDALERQYD---AFVAAQE
SGR_5536         MP-GHRSPFDEAQVPGSAEALVEYRLMESGHDVLGVATHVPHYVARSA-YPDAALTALEAITAATGLVLPALAHTLRTEAHRTQTEIDRQIGQGDEELVSLVEGLEHQYD----AVAGSE
AAur_1758        IE-GISSWKPTVDVPAAIGHILELRLTEAERNVAGYVIHVPHYLSEAE-YPPAAVAGLEYLGAATSLMLP--TDRLREAGREVGRQIAEQIEASEE-VQAVVTNLETRYD--EKSEGIVR
Mvan_2446        IA-EHQPWVNEVQVPGSASNLLEFRMAQHGYEVVGFTVHVPHYLAQTD-YPSAAETLLSEVARNGSLEIP--TTKLTQAAAEVFDKINEQVAGSAE-VAQVVEALERQYD---AFVAAQE
MUL_3357         IA-DFQPWISEIQVPASASNLLEYRMAQHGHEVVGFTVHVPHYLTQTD-YPAAAQALLEQVAKTGSLDLP--LAALTDASAQVGAKIDEQVQASAE-VAQVVAALERQYD---AFIDAQE
Rv2714           IS-DFQPSISEIQVPGSASNLLEYRMAQHGHEVVGFTVHVPHYLTQTD-YPAAAQALLEQVAKTGSLQLP--LAVLAEAAAEVQAKIDEQVQASAE-VAQVVAALERQYD---AFIDAQE
RHA1_ro06805     IK-DHQRWSGELQVPGSASSLIELRMAQHGHESVGFSVHVPHYLAQTD-YPGAAETLLENVSDVTDLDLP--LAALGEAAARVREQVDEHIAGNEE-VQTVVHALERQYD---AYVTAQE
Sare_1722        TA-GYEPWLQRVQVPGSVGYLLEYRLGEQGRDALGFAAHVPHYVAQTE-YPAAAEVLLSSVSRSTGLLLP--CDELRAATEAVRTEIDRQVAQTED-AAALVQALEEQYD--AFTRGRGQ
BLD_0447         EG-DR-----SYNGPVGIPTVLDVAAAQQGFAHSSMWVSIPQYLGSDE-CSAGTMRLLDALGKHIGFTFD--TADLKQKAEQWKAQASILVRCNDQ-LHDYVEHLEHDYDL--------
SSAG_03958       MP-GHRSPFDEAQVPGSAESLVEFRLGQAGHDVLGVAAHVPHYVARSP-YPDAALTALEAITAATGLVLPAVAHALRTEAHRTQTEIDRQIREGDEELVSLVQGLEHQYD----AAAGAE
BIFLAC_03242     DP-DY-----EYNGPIGITHALDMMAIEQGFNTTSLWVSIPRYLGSADPCPQATLTLLRELGEILGLDLP--AGTIENKAKQWRSRCDVTVRNNVD-FEHFVSQLERDYDL--------
Tfu_0926         VT-EHTPWVGRIQVPGNIMSLLEYRLGQAGHDVIGYAVHVPSYLAQSE-YPRAGLAVLEYIARVTGLVLP--TEKLAEEATRVDGEINRQV-EASEEVQRVVRNLEAQYDNFMAVHNGLD
SAV_6234         VP-GHRSPFEEAQVPGSAESLVEYRLMEAGHDVLGVAAHVPHYIARSP-YPDAALTVLEAITAATGLVLPGLAHSLRTEAHRTQTEIDRQIQEGDEELVALVQGLEHQYD----AAAGAE
BL0722           EG-DR-----SYNGPVGIPTVLDVAAAQQGFAHSSMWVSIPQYLGSDE-CSAGTIRLLDALGKYIGFIFD--TADLKQKAEQWKAQASILVRCNDQ-LHDYVEHLEHDYDL--------
Strop_1737       TA-GYEPWLQRVQVPGSIGHLLEYRLGEQGRDALGFAAHVPHYVAQTE-YPAAAEVLLASVSRSTGLLLP--SDGLRAATEAVRTEIDRQVAQTED-AAALVQALEEQYD--AFTRGRGQ
CMM_1644         ID-ALSVWKPHTQVPANALHLVEHRLHDAGHPVAGFVLLVPHYLADTE-FPLAAVAALESISAATGLIFP--TDRLREEGRDFVGRIDEQVAGNQE-LARLVTTLEERYD--SYMEDTPL
Francci3_1247    IV-GYEPWLRRLQVPGSAGHLLEFELGREGRDAMGFAAHVPHYLAQTT-YPAATEVLLTSVSKATGLLLP--LDGLRSAVAIQDEVDSQIARGGE-AAALVSALEEQYD--AYQRGRRG
MSMEG_2746       IA-EHTPWVGEVQVPASVSNLLEFRMAQHGHEVVGFTVHVPHYLAQTA-YPPAAEALLAEVARTGSLELP--LAALSEAGAEVYTKINEQVEASPE-VAQVVSALERQYD---AFIAAQE
ML1009           IA-DFTPWITEIQVPGSASNLLEYRMGQHGHEVVGFTVHVPHYLTQTD-YPAAAQALLEQVAKTGALQLP--LSALAEAAAEIRAKIDEQVQASTE-VAQVVAALERQYD---AFIDAQE
Acel_0028        IP-GNRPWLQQVHVPASAAHLLEFRLGEQGRKAMGIAVHVPHYVAQSE-FPPAALRLIDELRERTGLDLP--REPIATAAAATLAEIDRQVGESTD-AMAMVSTLEQQYD--AFLHARSG
FRAAL1976        VV-GYEPWLRRLQVPGSAGHLLEYELGRDGRDAMGFAVHVPHYLAQTT-YPAATEVLLTSVSKATGLMLP--LDGLRSAAVAVQDEVNSQIAQGGE-AAALVHALEEQYD--AYQRGRRG
Arth_1614        IE-GISVWKPTVEVPAAVGHILELRLSEAGRNIAGYVIHVPHYLAEAE-YPNAAVAGLEYLGAATSLMLP--TDRLREAGREVGRQIAEQLEASEE-VQQVVSRLETRYD--EKAEGTVR
Franean1_5208    LV-GYEPWVRRLSVPGSAGHLLEYEIGRSGADAMGFAAHVPHYLAQAT-YPAATEALLSSVSKSTGLLLP--LDGLRSAALEVRSEVDSQIARGGE-AADVVKAIEEQYD--AFHRGREG
KRH_14390        LP-RINGNRPTFEVSASVSHLLELRLQENGIDHVGLSVHVPQYLAESY-LPQAAVAALEYLSAVTGRTLP--TDELREASRDVIRQIDEQAGSSPE-VQAVIENMERRYD--DVVDASAR
BIFDEN_02062     EL-DK-----KYNGPIGIPNILDAIAADNGFSTTSMWVSVPQYFNNVE-CMQGTLELVHALSILLKQPLN--EGELANKAMQWRKEADDMVE-NMH-TGDYIRQLEREYDL--------
cu0890           VR-DYHTWDARLVVPGAAALETEMQLSAAGFDAIGLTAHVPHYIAGSE-YPEASYALLKAFADLADRELP--LKTLEKDMSRVQQQLAQQTQDSHE-IATVVGALEQQYD-KEVRRLQKK
jk1100           IQ-DFHTWDSRMIVPGAAALETEKKLNRHGFETVGLTAHVPHYIAASD-YPEAPLRLLQAFGKVANRELP--LKALEKELERVQHQLQDQVEDSQE-IATVVSALEHQYD-AEMERMRRK
NCgl1848         LK-DQVSLDTRMTVPGSASLMLEKLLKDKGKNVSGYTVHVPHYVSASP-YPAATLKLLQSIADSADLNLP--LLALERDAEKVHRQLMEQTEESSE-IQRVVGALEQQYD-SELERYRNR
cgR_1753         LK-DQVSLDTRMTVPGSASLMLEKLLKDKGKNVSGYTVHVPHYVSASP-YPAATLKLLQSIADSADLNLP--LLALERDAEKVHRQLMEQTEESSE-IQRVVGALEQQYD-SELERYRSR
MAP2831          IK-NFQPWIAEIQVPGSASNLLEYRMAQHGHEVVGYTVHVPHYLTQTD-YPAAAQALLEQVAKTASLELP--LTALSEAAEVIRAKIDEQVEASAE-VAQVVAALERQYD---AFIDAQE
MMAR_1998        IA-DFQPWISEIQVPASASNLLEYRMAQHGHEVVGFTVHVPHYLTQTD-YPAAAQALLEQVAKTGSLDLP--LAALTDASAQVGAKIDEQVQASAE-VAQVVAALERQYD---AFIDAQE
ACTODO_01571     LPPGSGAISNHMQFPSPLSTFMQIRMGQRGIGGLVLLGAVPYYMADTG-YPAASSALLTSFAKFADLSLP--VGDLEQGAAQDQENIAKLVEGNPD-ISHTVSNLEEHFDAWTGGEGAIP
MAV_3608         IK-NFQPWIAEIQVPGSASNLLEYRMAQHGHEVVGYTVHVPHYLTQTD-YPAAAQALLEQVAKTASLELP--LTALSEAAEVIRAKIDEQVEASAE-VAQVVASLERQYD---AFIDAQE
Mb2733           IS-DFQPWISEIQVPGSASNLLEYRMAQHGHEVVGFTVHVPHYLTQTD-YPAAAQALLEQVAKTGSLQLP--LAALAEAAAEVQAKIDEQVQASAE-VAQVVAALERQYD---AFIDAQE
CE1815           LK-SRFSIDARISIPGSASLKLEKLLHTRGKNVAGYTVHVPHYVAASP-YPAATLKLLQAVAETAELNLP--LLSIERDAEKVQRQLAEQTSDSME-IQHVVGALERQYD-EELERYREK
nfa31940         AE-RFNLEQTRYEGPTGITGVLQDQCVKAGVPAVSFWAAVPHYVSQPP-NPKATIALLHRVEDVLDIEVP--LGELPKQAEDWESAVSEMTEGDEE-ITEYVRSLEERGDA---------
RHA1_ro00860     AE-RFKLEQTRYEGPTGITGVLQDACVRAGVPAVSFWAAVPHYVSQPP-NPKATVALLQRVEDVLDIEVP--LGELPAQADDWQEAVTEMTADEE-IGEYVRSLEERGDA---------
Rv2125           AR-QFGLQETRYEGPTGIAGVFQSACVGAGIPAVTFWAAVPHYVSHPP-NPKATIALLRRVEDVLDVEVP--LADLPAQAEAWEREITETIAEDHE-LAEYVQTLEQHGDA---------
MtubH3_05282     AR-QFGLQETRYEGPTGIAGVFQSACVGAGIPAVTFWAAVPHYVSHPP-NPKATIALLRRVEDVLDVEVP--LADLPAQAEAWEREITETIAEDHE-LAEYVQTLEQHGDA---------
MT2184           AR-QFGLQETRYEGPTGIAGVFQSACVGAGIPAVTFWAAVPHYVSHPP-NPKATIALLRRVEDVLDVEVP--LADLPAQAEAWEREITETIAEDHE-LAEYVQTLEQHGDA---------
MUL_2357         AR-VFGLEETRYEGPTGIAGVFQYACVAAGIPAVTFWAAVPHYVSHPP-NPKATVALLRRVEDVLDIEVP--LSSLPAQAEVWEQEITEMTSDDDE-LAEYVQTLEKHGDE---------
Mvan_3475        AK-TFGLEETRYEGPTGIAGVFQDACVQAGIPAVTFWAAVPHYVSQPP-SPKATVALLRRVEDVLDIEVP--LADLPAQAEEWEQAVTEMTSEDDE-IAEYVQSLEERGDA---------
MAP1860          AK-RFGLEESRYEGPTGIAGVFQDACVAAGIPAVTFWAAVPHYVSQPP-NPKATIALLRRVEDVLDIEVP--LADLPTDAEEWEQAVTEMTAEDED-LAEYVQSLEQRGDA---------
MAB_2128c        AK-FFGLEETRYEGPTGIAGVFQDACVNAGIPAVAFWAAVPHYVSQPP-NPKATVALLQRVEDVLDVEVP--LGDLPEQAEEWEQAVTEMTTEDEE-IAEYVQSLEERGDA---------
Arth_2169        RE-RMNLEASQYEGPVGIVGVLSEVALLAGLPTVSLWAAVPHYVAQAP-SPKAQLALLHKIEELLQVPLD--THELAEEADAWERGVDELATEDPE-IAAYVRQLEEAKD---------
ML1306           AR-RFGLEETRYEGPTGIAGVFQDACVAARIPAVMFWAAVPHYVSHPP-NPKATVALLRRVEDVLDIEVP--LADLPTQAEDWEQAITEIAAEDDE-LAEYVHSLEQRGDA---------
Mflv_3052        AK-TFGLEETRYEGPTGIAGVFQDACVQAGIPAVTFWAAVPHYVSQPP-NPKATVALLRRVEDVLDIEVP--LADLPAQAEEWEQAVTEMTSEDDE-IAEYVQSLEERGDA---------
SGR_5841         AR-TMDLEETRYEGPTGIVGVLQEACTHAGVPAVSLWAAVPHYVSQPP-NPKATLALLNRLEDLIGLRIP--LGELPEDARAWQLGVDQLASEDSE-VAEYVQTLEEARD---------
RSal33209_2043   RE-RLNLEAPQYEGPIGIVGVLAEAAAGVGLPVLSLWAAVPHYVAQSP-SPKAQLALLHQLEELLQTPLE--TAEVVEEADAWERGVNELAVEDPE-IAVYVRQLEEAKD---------
SCO1662          AR-RMDLEETKYEGPTGIVGILAEGAECTHAGVPAVSLWAAVPHYVSQPP-NPKATLALLNRLEDLIGVRVP--LGELPEDARAWQVGVDQLAAEDTE-VAEYVQSLEEARD---------
Lxx08270         RA-ELGIERSSYEGPVGILSALAEGAEGVDIPTISIWASVPHYVHNAP-SPKAVLALIDKLEELVNVTIP--RGSLVEEATAWEAGIDALALDDDE-MATYIQQLEQARD---------
A20C1_02494      RT-ALGIERSSYEGPVGILSVLGQAAEKAGISTMSIWASVPHYVHNAP-SPKATLAIIDKLEEIVDVVIP--RGDLVTEATDWETGIDALAGEDDD-MSSYIEQLERARD---------
BlinB01000025    AT-HLGIDVNGYSGPIGILGLLGVECDRRGLRAINLWVGVPDYLTDSP-SPKAELALASAVEKYTGHEID--IPVLEEESRAWELGADELVALNPH-LVELLKGLEELNDS--------
SSAG_04705       AR-TMDLEETRYEGPTGIVGVLQEACTHAGVPAVSLWAAVPHYVSQPP-NPKATLALLNRLEDLIDIRIP--LGELPEDARAWQVGVDQLAAEDSE-VAEYVQTLEEARD---------
Rxyl_1077        VE-DLGLSASRYEGPTGITGVLHTLCAEAGLPAVSLWASMPHYLPAVP-SAPGALALVESLAGLLGTEVE--TGDLERGAERYQEQVSAAVARDPD-LSSYVKMLEERYDA--------
Noca_2630        VD-RLKLEQSTYEGPTGIVGVFQDACMRVDVPAVSYWAAVPHYVAQPP-CPKATLALIGQLEDLLEVSIP--LGDLPEDARAWERGVDELAQEDED-IADYVRALEETRD---------
TWT317           RY-EIGVAKPSYEGPVSVASAIQEAARKELIDTLSLWAPVPHYTQGLP-CPKALLALLVQVENLLSSSFTD-QISLKAEVQKWEERVTQAASSDPR-LLEYIKQLEELQDR--------
Mjls_3162        AK-FFGLVETRYEGPTGIAGVFQDACVQAGIPAVTFWAAVPHYVSQPP-NPKATVALLRRVEDVLDIEVP--LADLPTQAEEWEEAVTEMTAEDDE-IAEYVASLEQRGDA---------
MSMEG_4186       AK-FFGLEETRYEGPTGIAGVFQDACVQAGIPAVTFWAAIPHYVSQPP-NPKATVALLQRVEDVLDVEVP--LADLPQQAEEWEAAVTEMTAEDEE-IAEYVTSLEERGDA---------
Sare_2324        AH-RYQLTPTRYDGPTGIVGVLHDACAREAVDAVSFWVHVPHYANNPP-CPKATLALLHRVEEVLDLPVP--MADLAEEAAEWEQRVRGAAEQDAE-LGEYVRELEER-----------
FRAAL2860        AA-RLGLEPSRYEGPTGIVGVFGDACARAELASVSFWAAVPHYVANPP-CPKATLALLRRVEDLLDIEIP--LGDLPERAKAWERQVDELAAGDTE-VAEYVRSLESR-----------
SAV_6650         AR-TMDLEETKYEGPTGIVGILQEACTHAGVPAVSLWAAVPHYVSQPP-NPKATLALLNRLEDLIDVRIP--LGELPEDARAWQVGVDQLAAEDSE-VAEYVQSLEEARD---------
Strop_2170       AQ-RFQLTPTRYDGPTGIVGVLHDACSRAEVDAVSFWVHVPHYANNPP-CPKATLALLHRVEEVLDLPVP--MADLAEEAAEWEQRVRGAAEQDAE-LGEYVRELEER-----------
Francci3_2640    AA-RLGLEPSRYEGPTGIVGVFGEACARAELASVSFWAAVPHYVANPP-CPKATLALLRRVEDLLDIEIP--LGDLPERAKAWERQVDELAAGDTE-VAEYVRSLESR-----------
Acel_1173        AR-RLGLEQSHYEGPTGIVGVFNDACIQAGLPSVSLWAAVPHYVASPP-CPKATLALLRHLEVVLDIPMP--YGDLPELAIAWERGVDQLAEEDSE-IAEYVRSLEQQKD---------
Tfu_1826         AT-SASLEPATYEGPTGIVGVLHDIFSAAGLETISLWAAVPHYVSQPP-SPKATLALVRRVEDTLDLTVR--LGELPENARAWERGVDELAAQDTD-IADYVRSLEEAKD---------
Krad_1842        AE-KLDVEPSRYEGPTGIVGVVQDAVATAGIASLSLWAAIPHYVGQPP-SPKGTLALLRRVEELLDLAVP--LADLPEDARAWERGVDELAAEDSE-IAEYVQQLEEAKD---------
KRH_13910        RT-ELDIEAPQYEGPTGIVGVLSHMSALAGFPTLSMWAAVPHYVAHSP-SPKAQLALARQLEDLLGMSLP--LDPVVEDAEAWERGVDELASEDPE-VADYVRQLEEAKD---------
Franean1_4890    AA-RLGLEASRYEGPTGIVGVFADACSRAELASVSFWAAVPHYVANPP-CPKATLALLRRVEDLLDIEIP--LGDLPDKAKAWERQVDELAAGDTE-VAEYVRSLESR-----------
SACE_2233        AT-RYGLEQSKYEGPTGIVGIFQDACVQAGIPAISFWAAVPHYVSQPP-SPKATLALLHRVEEALDLEVP--LGALPEQAEEWETTVSEMAEEDED-VRNYVRALEERGDA---------
JNB_03080        RH-RHDLEASTYEGPTGIVGVISDAAIQVGISSISVWAAVPHYAHHTP-SPKATRAIVSRLEELLDGTID--HEGLDDDARAWENGLDALASEDSE-VAEYVESLEEAQD---------
CMM_1693         RA-ELGIERSTYEGPVGILSIIAQRAEEMGMPTVSLWASVPHYVHSAP-SPKATLALIDKLEEMVDVVIP--RGELIQEAATWEAGIDQLAGEDED-MASYIQQLEQARD---------
CMS_1670         RA-ELGIERSTYEGPVGILSIIAQRAEEMGMPTVSLWASVPHYVHSAP-SPKATLALIDKLEEMVDVVIP--RGELIQEAATWEAGIDQLAGEDED-MASYIQQLEQARD---------
PPA1200          IS-ELGAEKLEYDGPTGIPFVFVTEARRHGMNAIGLWVGVPQYACESL-CPPAVQALLVRIEELTGIAIP--QAGLEEDKRDWVAAIDEALVDHPE-LAEYIAALETEQDA---------
AAur_2170        RE-RLNLEASQYEGPVGIVGVLAEFAMLAGLPTVSLWAAVPHYVAQPP-SPKAQLAIIHRIEDLLQVPLD--SQALAEESAEWERGVDELATEDPE-ITAYVRQLEEAKD---------
TW455            RY-EIGVAKPSYEGPVSVASAIQEAARKELIDTLSLWAPVPHYTQGLP-CPKALLALLVQVENLLSSSFTD-QISLKAEVQKWEERVTQAASSDPR-LLEYIKQLEELQDR--------
BIFADO_01161     ES-DR-----EYNGPVGIPNILDAFAAEAGFPTTSIWVSVPHYCANTE-CMQGTLELLRALSLIIEYPLI--EGDLTRKAMQWRSDADGLVS-DMH-AGDYLARLEHDYDL--------
BAD_0824         ES-DR-----EYNGPVGIPNILDAFAAEAGFPTTSIWVSVPHYYANTE-CMQGTLELLRALSLIIEYPLI--EGDLTRKAMQWRSDADGLVS-DMH-AGDYLARLEHDYDL--------
                                      .                  :* *              .     .       .     :                            :  :*
```

```
SCO1997        TRG-N--MLAEPVEIPSADEIGREFERFLAE----------------------------REGDG------------------------------------------------
RSal33209_2429 RSL-L--AG-DGDELPDADELGAAIEAYLAER---------------------------DDTAERDDTTG------------------------------------------
MAB_3033       NRS----LLARDEELPSGDELAGEFERFLAEQAKFG------------------------DDPSGPSPEGPIL---------------------------------------
Noca_2852      SGT-S--LLARDQRLPTGEEIGKEFERFLAGLDRPG------------------------DDEPQG----------------------------------------------
A20C1_03668    RSP-L--TD-EDGELPSADEIAAELQKFLAIR---------------------------RAGDDDSTTGLPL----------------------------------------
Krad_1509      -------TGAASAFLPTADELGEEFERFLAGQE--------------------------DEPDGDGPPGDRPGAG-------------------------------------
CMS_1627       KSP-L--TD-EDGALPTADEIAAELEKFLARR---------------------------RPGDGDAA---------------------------------------------
Lxx10170       RSP-L--TD-RDGELPSADEIAAELENFLAFR---------------------------RHGDEDNSRGDR-----------------------------------------
nfa37830       RQS-S--LLAADGDLPSGEELGAEFERFLAEQGGYDG-----------------------DKD-------------------------------------------------
PPA1099        -------LGTTQVAVPDAEDIGAEVEDFLRSIDEDDGPSN-------------------DDPDTQGSGPRRSSDDTSDDDKPDYHPRRGGDLDN------------------
JNB_00545      RPS-L--LATEAEDLPSADELGADIEEFLRNVSDD-----------------------------------------------------------------------------
DIP1417        NPK-A--LEIAEDDLPDADELGEEFERFLANIDEEN----------------------GSDD--------------------------------------------------
Mflv_3951      NRS----LLARDEDLPSGEELGAEFERFLAQQAGEKKRKD-------------------GDDQPREGL--------------------------------------------
SGR_5536       TRG-N--LVAEPVDLPSADEIGREFERFLAE----------------------------REGEG------------------------------------------------
AAur_1758      RSL-L--AD-ENDELPNAEDIGAAVEAYLARK---------------------------DSAQ-------------------------------------------------
Mvan_2446      NRS----LLARDEDLPSGEELGAEFERFLAQQAGEKKRKD-------------------GDD-PREGL--------------------------------------------
MUL_3357       NRS----LLTRDEDLPSGDELGAEFERFLAQQAEKK-----------------------FDDDDQP----------------------------------------------
Rv2714         NRS----LLTRDEDLPSGDELGAEFERFLAQQAEKK-----------------------SDDDPT-----------------------------------------------
RHA1_ro06805   QQS-T--LLASEEDLPSGDELGAEFERFLAEQALQDGEG--------------------GDSDDEGGSPGGA----------------------------------------
Sare_1722      PNL-L--NTG-AGSLPTADELGAELERFLAEQ---------------------------TRPND-NPGG-------------------------------------------
BLD_0447       ---QQKAEAEASLGAPQAEQLVKEAEAFLRQMGN------------------------------------------------------------------------------
SSAG_03958     TRG-N--MLAEPAEIPSADEIGREFERFLAE----------------------------REGEG------------------------------------------------
BIFLAC_03242   ---HQHAKELSSAGTPMAEQLVQEAEAFLRDQD-------------------------------------------------------------------------------
Tfu_0926       VDA-L--LAGDESRLPTADELGAELERFLAEHGRGSAEHD-------------------RGSEG------------------------------------------------
SAV_6234       TRG-N--MLAEPVEIPSADEIGQEFERFLAE----------------------------REGEG------------------------------------------------
BL0722         ---QQKAEAEASLGAPQAEQLVKEAEAFLRQMGN------------------------------------------------------------------------------
Strop_1737     PNL-L--STG-TEALPTADELGAELERFLAEQ---------------------------TRPND-NPGG-------------------------------------------
CMM_1644       KSP-L--TD-EDGALPTADEIAAELEKFLARR---------------------------RPGDGDAA---------------------------------------------
Francci3_1247  PSL-P--AADDVQPLPTADELGDALERFLAEQ---------------------------TEPDGPTPN--------------------------------------------
MSMEG_2746     NRS----LLARDEDLPSGDELGAEFERFLAQQAGEKFKDDKYKDGHGEGFGGRIRGTVTTTDDVPDQG--------------------------------------------
ML1009         NRS----LLRRDEDLPSGDELGAEFERFLAQQAEKK-----------------------RDDDLT-----------------------------------------------
Acel_0028      ESP-L--GGG-TDNLPSADELAAELERFLAEQ---------------------------ARRHRDDRDQ-------------------------------------------
FRAAL1976      PSL-P--TIDAEQKLPTADELGEALERFLAEQ---------------------------SEPGGPNPLS-------------------------------------------
Arth_1614      RSL-L--AD-ENDELPNADALGAAVEAYLARE---------------------------EPRQ-------------------------------------------------
Franean1_5208  EHL-P--VVDDSEPLPTGEELGAALERFLAEQ---------------------------SEPGGPQPT--------------------------------------------
KRH_14390      RSI-L--AGPDDADLPDADDLGAAAEAFLAAQ---------------------------SDPDAHED---------------------------------------------
BIFDEN_02062   ---NVKAREIASNGTPACEQLIQEAEAFLRNQPFAEH---------------------------------------------------------------------------
cu0890         K-S-S--LLEPGQDIPTGEELGAEFEAFLARMNDAA----------------------DTDSEADGEDPKKPGDSNDPHDDGEGDTQ-------------------------
jk1100         REN-N--LLKPGQDIPTGEELGAEFEAFLQNMSDGD----------------------LSD--ENGADN--SADSDEQEEN-------------------------------
NCgl1848       HPQ-A--VMPGESELPSGDEIGAEFEKFLADLDDQG----------------------GSDHKETPEA--------------------------------------------
cgR_1753       HPQ-A--VMPGESELPSGDEIGAEFEKFLADLDDQG----------------------GSDHKETPEA--------------------------------------------
MAP2831        NRS----LLTRDGDLPSGDELGAEFERFLAQQAEKK-----------------------FDDDD-QA---------------------------------------------
MMAR_1998      NRS----LLTRDEDLPSGDELGAEFERFLAQQAEKK-----------------------FDDDDQP----------------------------------------------
ACTODO_01571   LPG-LGQRPSAGGEEKTPKDIGDVIEAYLAQVSRAQDEEIESVQR-----------APRTEESEEPSKPDTIEDVLARIEARRNGKGTGPSAPRHRA---------------
MAV_3608       NRS----LLTRDGDLPSGDELGAEFERFLAQQAEKK-----------------------FDDDDDQA---------------------------------------------
Mb2733         NRS----LLTRDEDLPSGDELGAEFERFLAQQAEKK-----------------------SDDDPT-----------------------------------------------
CE1815         NPQ-A--VLPGETPVPSGDEIGAEFERFLANLDDSG----------------------DPDGPEDNGQ--------------------------------------------
nfa31940       -----AVDMKEAIAKIDGDAIAAEFEKYLRRRGPGSFGL-------------------------------------------------------------------------
RHA1_ro00860   -----ETDISDAISKIDGDALAAEFERYLRRRGPGSFGL-------------------------------------------------------------------------
Rv2125         -----AVDMNEALGNIDGDALAAEFERYLRRRRPG-FGR-------------------------------------------------------------------------
MtubH3_05282   -----AVDMNEALGNIDGDALAAEFERYLRRRRPG-SGARGRLRCGGRRCQPGGPGSPESP---------------------------------------------------
MT2184         -----AVDMNEALGNIDGDALAAEFERYLRRRRPG-FGR-------------------------------------------------------------------------
MUL_2357       -----AVEMNDALGKIDGDALAAEFERYLRRRRPG-FGL-------------------------------------------------------------------------
Mvan_3475      -----EVDMNEALGKIDGDALAAEFERYLRRRGPG-FRG-------------------------------------------------------------------------
MAP1860        -----EVDMNDALGKIDGDALAAEFERYLRRRRPG-FGR-------------------------------------------------------------------------
MAB_2128c      -----EVDTTEMLSKIDGDALAAEFERYLRRRGPR-FGG-------------------------------------------------------------------------
Arth_2169      ---------TADLPEASGESIAREFERYLKRRGKDKQ---------------------------------------------------------------------------
ML1306         -----EVDVNDALGKIDGDALAAEFERYLRRRRPG-FGR-------------------------------------------------------------------------
Mflv_3052      -----EVDMNEALGKIDGDALAAEFERYLRRRGR------------------------------------------------------------------------------
SGR_5841       ---------TAELPEASGEAIAREFERYLRRRDGGPG---QGPGGHATESGD-----IPYLRDPATGRTRPPKP-PRTEPGRGRDADGQGAEGPDETPGKDPGNGSETPDED
RSal33209_2043 ---------TAELPEATGESIAREFERYLRRRGKEKP---------------------------------------------------------------------------
SCO1662        ---------TAELPEASGEAIAREFERYLRRRDGAGPGGPGGPGGHATADGGDGPAGAPFRRDNPSGRARPPKP-----PKAGGDDE--------ESSED------------
Lxx08270       ---------TVDSPEASGEAIAQEFERYLRRRD-----GRAGDD-PRRG---------------------------------------------------------------
A20C1_02494    ---------TVDSPEASGEAIAQEFEKYLRRGDDKPDQGSAGDE-PWRGKD-------------------------------------------------------------
BlinB01000025  ----------TELPEASGDAIAAEFERYLRKRGRER----------------------------------------------------------------------------
SSAG_04705     ---------TADLPEASGDAIAREFERYLRRRD--P----AGPS--STEPGD-----GSYLRDTSGGLPRPPMRRPDPEPGRRVRARSRTIR----TPRR------------
Rxyl_1077      ---SQEEGGGQIRHLPSGDELARELEDFLRRQRGEDEQ--------------------------------------------------------------------------
Noca_2630      ---------TTDLPEASGEAIAREFERYLKRRQGDE----------------------------------------------------------------------------
TWT317         ----------VALSSTANDHLVKSLERYLSSIRRESEEFPTISRLRELPE--------------------------------------------------------------
Mjls_3162      -----EVDMTEVLGKIDGDALAAEFERYLRRRGPG-FRS-------------------------------------------------------------------------
MSMEG_4186     -----EVDLHEVLGKIDGDALAAEFERYLRRRGR------------------------------------------------------------------------------
Sare_2324      -------VGDAGITPLTGDEIAQEFEKYLRRRGGSAGPTAGSW---------------------------------------------------------------------
FRAAL2860      -------EPETALPEASGDAIAREFERYLRRRGQ------------------------------------------------------------------------------
SAV_6650       ---------TAELPEASGEAIAREFERYLRRRDGGGP----AAGGHATADGSDGTS---YLRDAPGGRTRPPKP-----PKPEAEDDGPADEGSDDSSED------------
Strop_2170     -------VGDAGITPLTGDEIAQEFEKYLRRRGGSAGPTAGSW---------------------------------------------------------------------
Francci3_2640  -------EPETALPEASGDAIAREFERYLRRRGQ------------------------------------------------------------------------------
Acel_1173      ---------DDDVPEGAGEAIAREFERYLRHRREGQS---------------------------------------------------------------------------
Tfu_1826       ---------AAELPEASGEAIAREFERYLRRRRGGS----------------------------------------------------------------------------
Krad_1842      ---------TAELPEASGEAIAREFERYLRRRDDPPRRG-------------------------------------------------------------------------
KRH_13910      ---------TVDLPEATGDAIAREFERYLRRRDDRG----------------------------------------------------------------------------
Franean1_4890  -------EPETALPEASGDAIAREFERYLRRRGE------------------------------------------------------------------------------
SACE_2233      -----EVTLTETS----GDAIAAEFERYLRRRGPGGKGPKGPGPG-------------------------------------------------------------------
JNB_03080      ---------TAELPEASGDAIAKEFERYLRKRGTDD----------------------------------------------------------------------------
CMM_1693       ---------TVDSPEASGEAIAQEFEKYLRRGDGPRGRGDDRPEEPWRPKDPKDPKDQ------------------------------------------------------
CMS_1670       ---------TVDSPEASGEAIAQEFEKYLRRGDGPRGRGDERPEEPWRPKDPKDPKDQ------------------------------------------------------
PPA1200        ----------ALSEGLTGDVLAVEFQSYLRHRGH------------------------------------------------------------------------------
AAur_2170      ---------TADLPEASGESIAREFERYLKRRGKERP---------------------------------------------------------------------------
TW455          ----------VALSSTANDHLVKSLERYLSSIRRESEEFPTISRLRELPE--------------------------------------------------------------
BIFADO_01161   ---DARAKRIASNGMPACEELLREAESLLRNGV-------------------------------------------------------------------------------
BAD_0824       ---DARAKRIASNGMPACEELLREAESLLRNGV-------------------------------------------------------------------------------
                                 . :    :  *
```

Note: Conserved residues colored in Additional file 2 are also highlighted here. Unexpected amino acids at conserved positions are highlighted in cyan.
